# Variation in gut bacterial composition is associated with *Haemonchus contortus* parasite infection of sheep

**DOI:** 10.1186/s42523-020-0021-3

**Published:** 2020-02-05

**Authors:** Md. Abdullah Al Mamun, Mark Sandeman, Phil Rayment, Phillip Brook-Carter, Emily Scholes, Naga Kasinadhuni, David Piedrafita, Andrew R. Greenhill

**Affiliations:** 1 Monash University, Faculty of Science, Melbourne, VIC 3800 Australia; 2 Animal Health, Ecology and Diagnostics Research Group, School of Health and Life Sciences, Federation University Australia, Gippsland Campus, Northways Rd, Churchill, 3842 Australia; 3 Dept of Parasitology, Bangladesh Agricultural University, Mymensingh, 2202 Bangladesh; 4 Australian Genome Research Facility, Melbourne, QLD 4072 Australia

**Keywords:** Worm parasite, Ruminant, Protective effect, Gut bacteria, Firmicutes

## Abstract

**Background:**

One of the greatest impediments to global small ruminant production is infection with the gastrointestinal parasite, *Haemonchus contortus*. In recent years there has been considerable interest in the gut microbiota and its impact on health. Relatively little is known about interactions between the gut microbiota and gastrointestinal tract pathogens in sheep. Thus, this study was undertaken to investigate the link between the faecal microbiota of sheep, as a sample representing the gastrointestinal microbiota, and infection with *H. contortus*.

**Results:**

Sheep (*n* = 28) were experimentally inoculated with 14,000 *H. contortus* infective larvae. Faecal samples were collected 4 weeks prior to and 4 weeks after infection. Microbial analyses were conducted using automated ribosomal intergenic spacer analysis (ARISA) and 16S rRNA gene sequencing. A comparison of pre-infection microbiota to post-infection microbiota was conducted. A high parasite burden associated with a relatively large change in community composition, including significant (*p* ≤ 0.001) differences in the relative abundances of Firmicutes and Bacteroidetes following infection. In comparison, low parasite burden associated with a smaller change in community composition, with the relative abundances of the most abundant phyla remaining stable. Interestingly, differences were observed in pre-infection faecal microbiota in sheep that went on to develop a high burden of *H. contortus* infection (*n* = 5) to sheep that developed a low burden of infection (*n* = 5). Differences observed at the community level and also at the taxa level, where significant (*p* ≤ 0.001) in relative abundance of Bacteroidetes (higher in high parasite burden sheep) and Firmicutes (lower in high parasite burden sheep).

**Conclusions:**

This study reveals associations between faecal microbiota and high or low *H. contortus* infection in sheep. Further investigation is warranted to investigate causality and the impact of microbiome manipulation.

## Background

Growing populations and incomes, along with changing food preferences, are rapidly increasing demand for livestock products. But there are obstacles to meeting projected global demands, among them parasite infection is considered one of the major impediments in the livestock industry.

Haemonchosis, the clinical disease caused by infection with *Haemonchus* spp., infects goats, sheep and cattle in tropical and sub-tropical regions [1]. A burden of 1000 parasites can cause acute anaemia in small ruminants, and can be fatal if untreated, especially in young sheep where immunity is less developed than in adult sheep [2]. Infections negatively impact animal production due to concomitant reductions in milk, wool and meat production, reduced reproductive performance, sudden death of animals and cost of on-going drug treatment [3, 4].

Over the past 15 years there has been an explosive growth in analysis of microbial populations through genetic amplification and detection technologies; with the hope that research in this field can translate to improved health. To date, much of the work conducted has been in humans [5–7], though numerous studies have investigated the microbial composition of the digestive tract of other animals, including production animals [8–10]. The importance of microbes in the digestive tract of animals has long been appreciated, particularly their role in the digestion of cellulose in ruminants. However, recent research has revealed that microbes in the digestive tract play a role in many aspects of an animal’s physiology, including proper development of intestinal morphology and digestive function, as well as immune function [11–14]. Moreover, intestinal microbes are thought to greatly influence the development and effectiveness of mucosal and systemic immune responses in mammalian systems [15].

*H. contortus* infects the abomasum (true stomach) of ruminants and activates numerous biological pathways, including immune-mediated pathways in the mucosa of the pyloric abomasum [16] which are likely to interact and modulate the resident microbiota: although this is a poorly defined area of research. In addition, infection is likely to have an impact on the gut microbiota as the parasite causes serious physiological changes within the abomasum after infection, namely increases in the abomasal pH and a decrease in the abomasal *P*O2 [16, 17]. Infection with enteric parasites affects the gut microbial population in different host animals, including ruminants [16, 18–27]; however, to date little research has been conducted on microbial populations in association with *H. contortus* infection. We hypothesised that the gut bacterial populations might have impact on the burden of parasite infection. Therefore, the objectives of this study are to a) better understand the interactions between *H. contortus* infection and gut microbial composition; and b) explore relationships between gut microbiota and severity of *H. contortus* infection.

## Results

### Burden of parasite infection

**Fig. 1:**
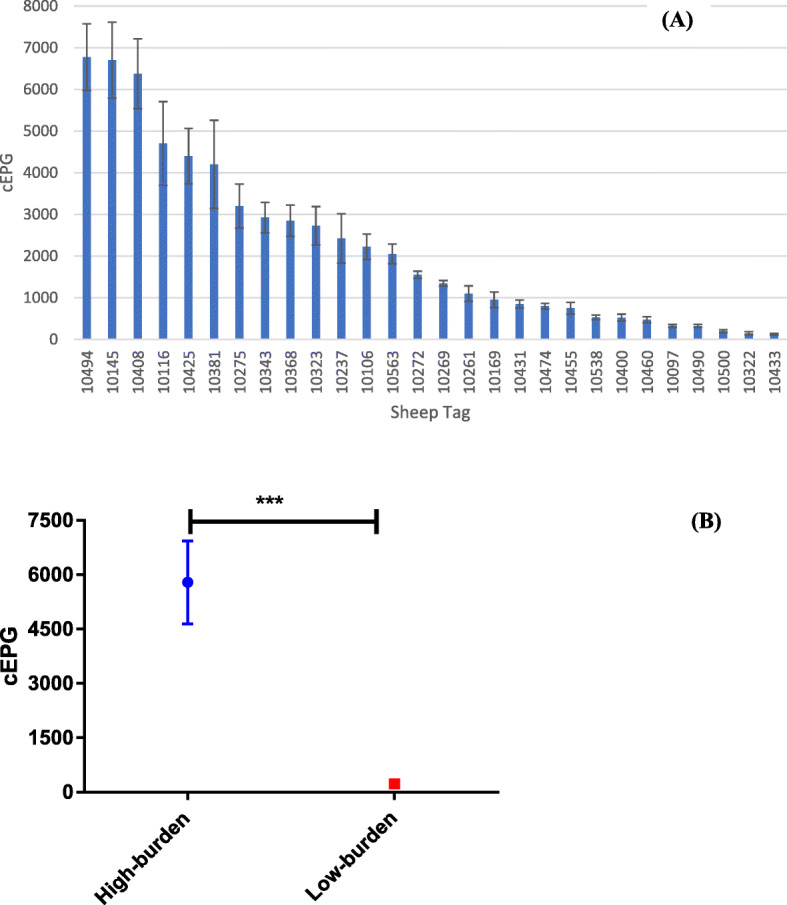
Distribution of egg count data. (**a)** cEPG for each of the 28 sheep. (**b**) Comparison of cEPG of high (*n* = 5) and low (*n* = 5) parasite burden sheep. Unpaired t-test. Error bars represent SD. ***p ≤ 0.001

Cumulative faecal egg count (cFEC) data obtained 3–5 weeks post infection (# egg counts) were used to rank sheep according to burden of infection (Fig. 1a). Based on this ranking, faecal samples from 10 selected sheep (5 sheep with the highest cFEC to the 5 sheep with the lowest cFEC) were used for further analyses, and are referred to as high burden (*n* = 5) and low burden (*n* = 5) sheep. There was a significant difference (*p* ≤ 0.001) in the worm burden (mean ± SD) of the two groups (high burden sheep: 5790 ± 1146.81; low burden sheep: 225 ± 95.19) (Fig. 1b).

### Community structure of faecal microbiota of high- and low-burden sheep prior to infection

**Fig. 2:**
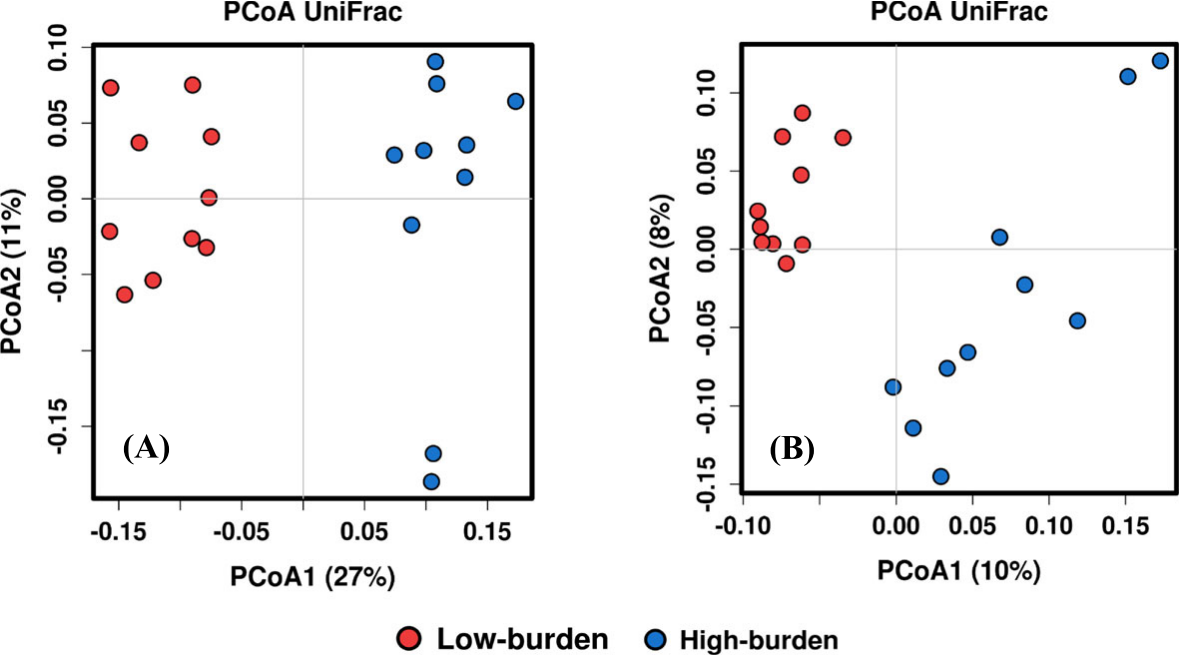
Distinct clustering of microbiome between high and low worm burden sheep using 16S data. PCoA plot of sheep based on weighted UniFrac (**a**) and un-weighted UniFrac (**b**) distance of faecal microbial composition. Each symbol represents an individual sheep sample

A total of 4,264,934 (106,623 ± 31,466; mean ± SD) sequences were obtained from the hyper variable V1-V3 region of the bacterial 16S rRNA gene. After quality control (QC) and chimera removal, samples contained a total 3,210,471 sequences; an average of 80,262 ± 23,384 (mean ± SD) sequences per sample. This resulted in a total of 11,732 OTUs at ≥97% sequence similarity. Rarefaction analysis based on the OTU richness values (Additional file 1: Figure S1) suggested that sequencing depth for this study was adequate. Multivariate ANOSIM analysis based on Bray-Curtis similarities, and weighted and unweighted UniFrac distance, revealed a separation of microbial composition between the sheep with high and low parasite burden (Additional file 1: Table S1). A PCoA plot of 16S sequence data (Fig. 2) showed two distinct clusters of faecal microbiota, corresponding with high-burden and low-burden sheep. Microbial diversity indices were evaluated (Table 1). Species richness and Fisher’s alpha indices were significantly (*p* ≤ 0.05) different between high-burden and low-burden sheep with low-burden sheep having higher diversity. Other indices were not significantly different.

**Table 1:** Microbial diversity indices of the faecal microbiota of high and low parasite burden sheep

Diversity index	High-burden (mean ± SD)	Low-burden (mean ± SD)	*p* value
Species richness	2335.5 ± 42.33	2383.4 ± 30.10	0.03*
Species evenness	0.88 ± 0.005	0.88 ± 0.004	0.22
Chao1	3849.39 ± 101.84	3926.63 ± 86.53	0.14
Fisher’s alpha	956.86 ± 26.76	984.46 ± 26.06	0.03*
Shannon	6.83 ± 0.04	6.86 ± 0.03	0.13
Simpson	0.997 ± 0.0003	0.997 ± 0.0002	0.31

Significance determined using the Mann Whitney test

*p ≤ 0.05

### Community composition of faecal microbiota of high- and low-burden sheep prior to infection

**Fig. 3:**
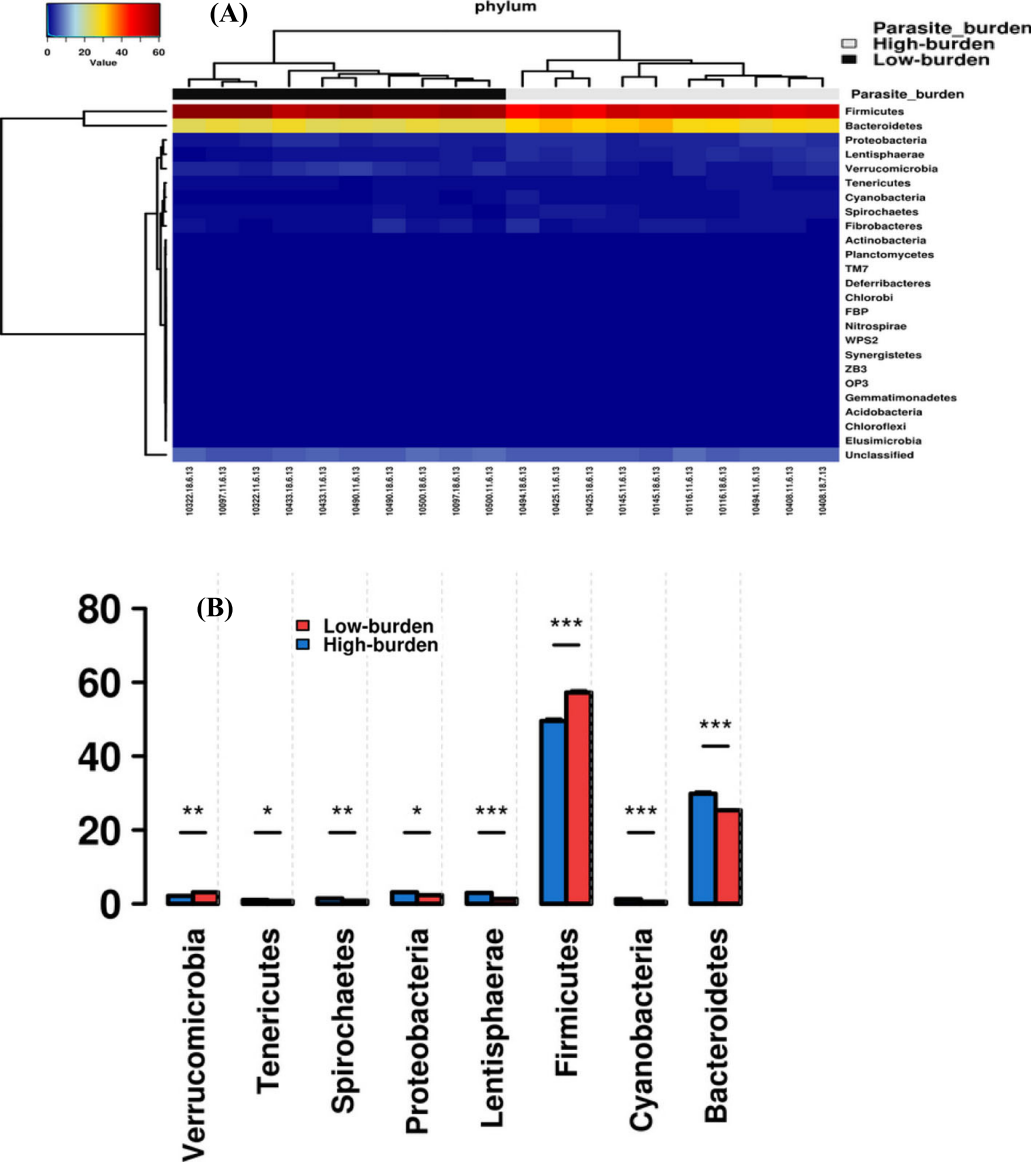
Average relative abundances (%) of identified phyla. (**a**) HeatMap+ of the relative abundances of the identified phyla; (**b**) the phyla with a significant difference in relative abundances in high and low burden sheep. The Y-axis shows the average relative abundances (%); X-axis shows phyla. Unpaired t-test. Error bars represent SD. *p ≤ 0.05, **p ≤ 0.01, ***p ≤ 0.001

16S rRNA gene sequence data revealed the gut microbiota of sheep was dominated by 24 bacterial phyla; with eight phyla having significantly (*P* ≤ 0.05) different relative abundances in high-burden sheep relative to low-burden sheep (Fig. 3b). Notably, there were significantly (*p* ≤ 0.001) more Firmicutes and less Bacteroidetes in low-burden sheep relative to the high-burden sheep. Of 133 families identified, 25 families had significantly different abundances in the two aforementioned groups of sheep (Additional file 1: Figure S2). At genus level, 36 out of 200 genera identified had significantly different (*p* ≤ 0.05) abundances in the two groups of sheep (Additional file 1: Table S2).

**Fig. 4:**
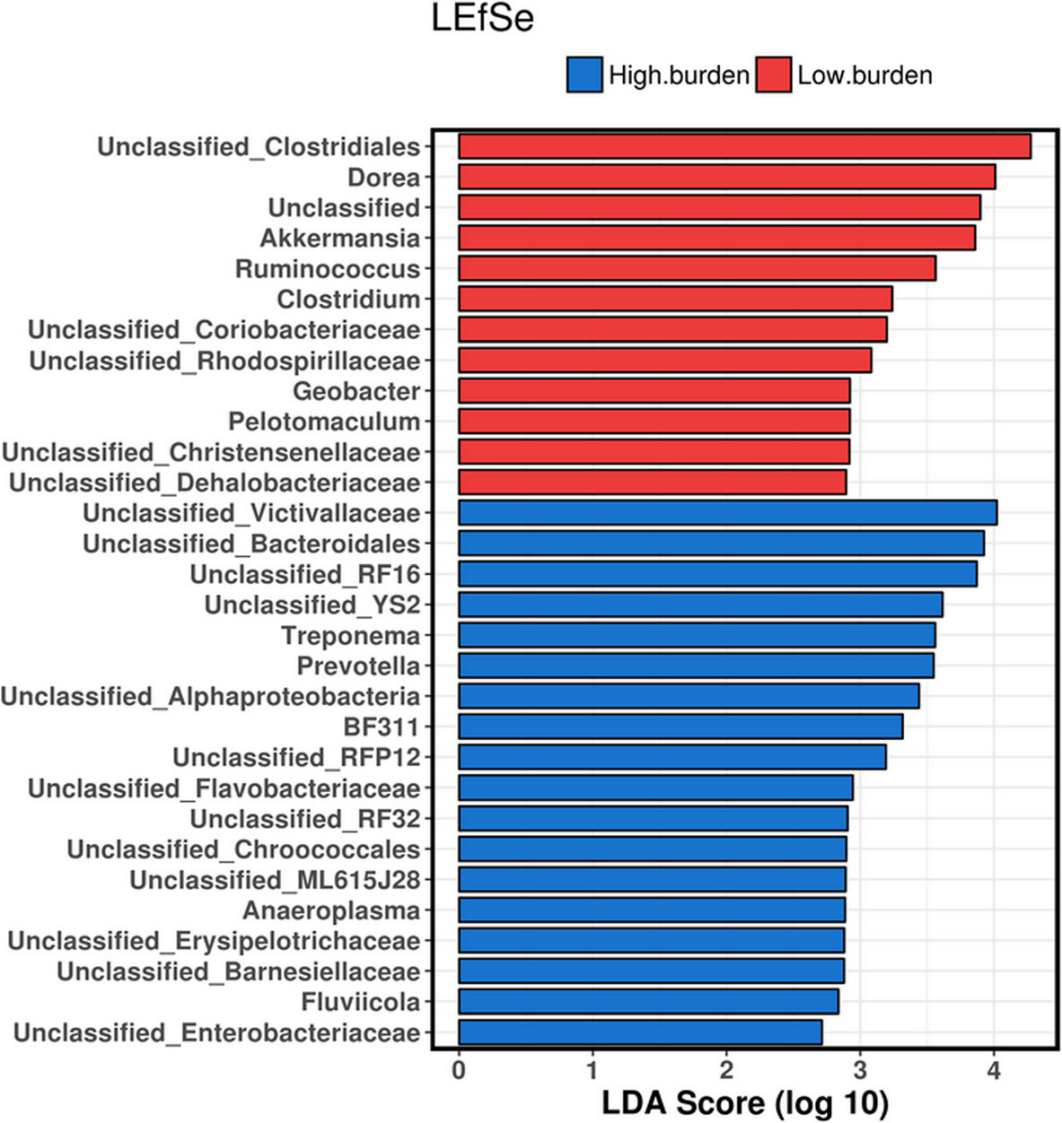
Genera with absolute LDA score ≥ 2.0. Taxa (genus) associated with the differences between high and low burden sheep (before infection) were identified using LEfSe

Of the 200 OTUs identified to genus level, 30 had significant differences in relative abundances in high-burden sheep relative to low-burden sheep using the stringent cut-off value of absolute LDA score log10 ≥ 2.0 in LEfSe (Fig. 4). Among these genera, *Treponema* and *Prevotella* were associated with high-burden sheep; whereas *Dorea*, *Clostridium* and *Akkermansia* were found to be more prevalent in low-burden sheep. Additionally, a number of unclassified genera were highly abundant in high and low burden sheep.

Approximately 60% of OTUs were present in a minimum 10% of the samples of both high-burden and low-burden groups (Additional file 1: Figure S3), whereas 55.04% core OTUs (present in minimum 50% of the samples within the group) were shared between the two groups (Additional file 1: Figure S4) but their abundance differed (Additional file 1: Table S3).

### Community structure of faecal microbiota of high- and low-burden sheep before and after *H. contortus* infection

**Fig. 5:**
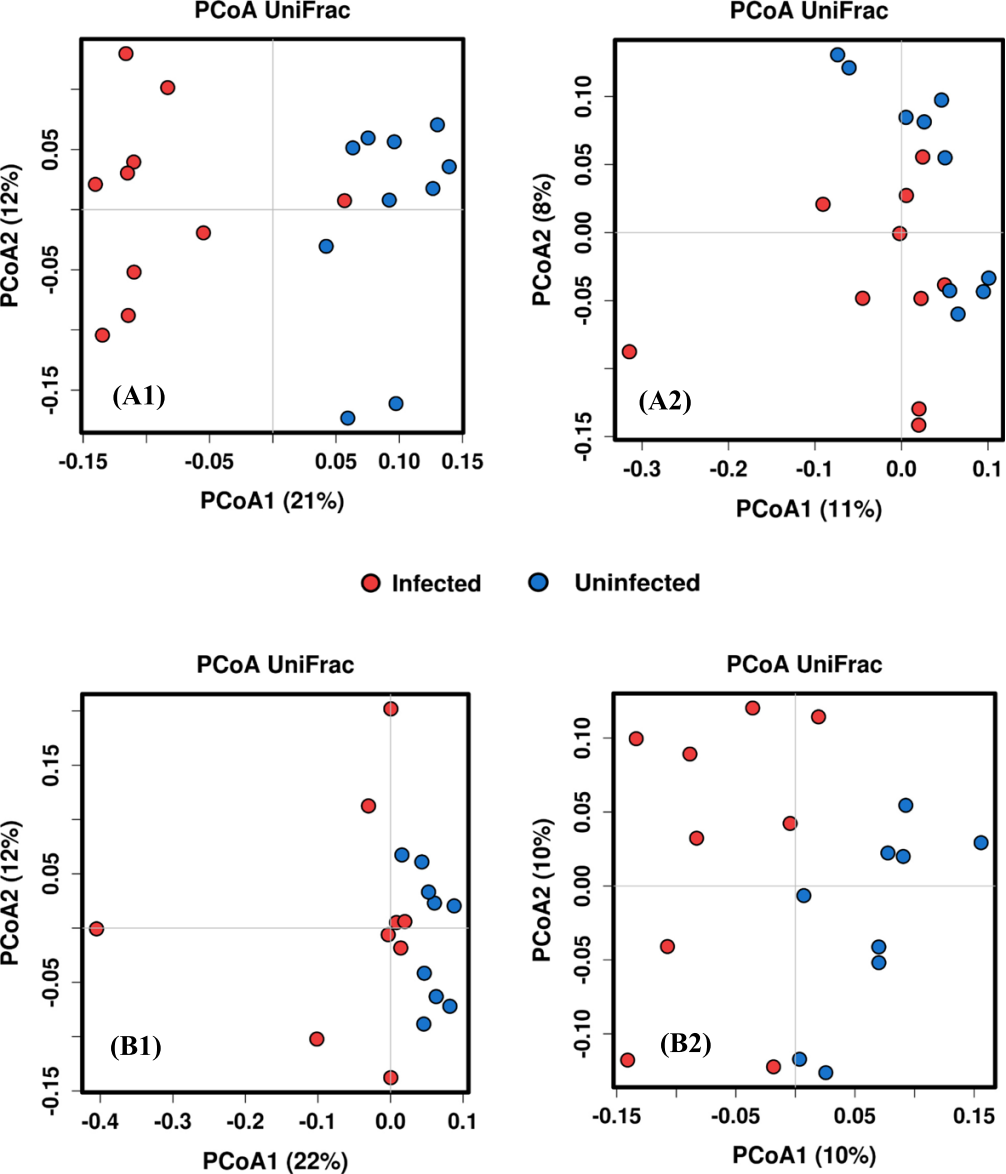
Clustering of microbiome of infected and uninfected sheep based on 16S data**.** (**a**) high-burden; (**b**) low-burden sheep. PCoA plot of sheep based on weighted (A1 and B1) and unweighted (A2 and B2) UniFrac distance of gut bacterial composition. Each symbol represents an individual sheep sample

**Fig. 6:**
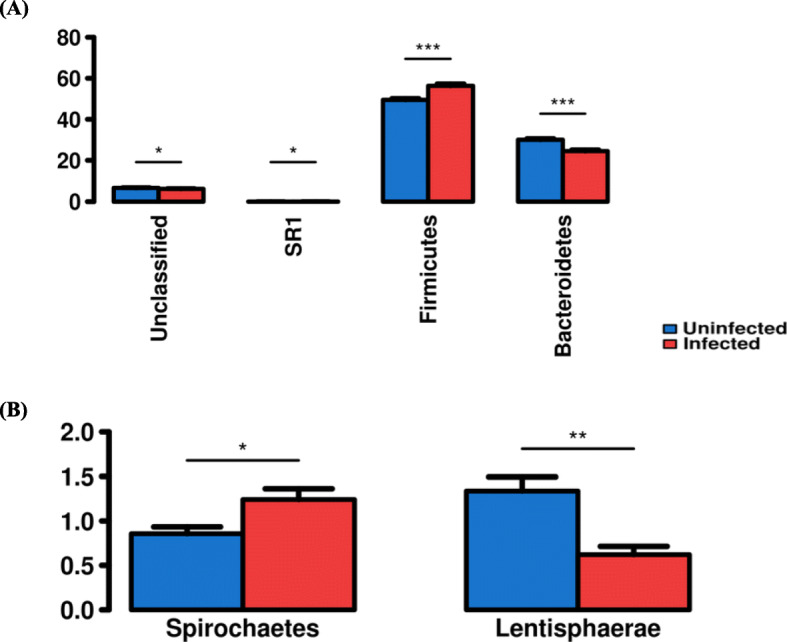
The bacterial phyla with significant differences in relative abundances before and after infection. (**a**) high-burden; (**b**) low-burden sheep. The Y-axis shows the average relative abundances (%); X-axis shows phyla. Pair-wise comparisons are done by paired t-test. Error bars represent SD. *p ≤ 0.05, **p ≤ 0.01, ***p ≤ 0.001

ANOSIM analysis based on Bray-Curis similarities and weighted UniFrac distance of 16S sequence data revealed separation of faecal microbial composition in sheep with high worm burden before infection relative to after infection (Additional file 1: Table S4). In sheep with low parasite burden a comparatively smaller separation was detected (Additional file 1: Table S4). PCoA plot based on the weighted UniFrac distance showed two distinct clusters in high-burden sheep: one for uninfected sheep and the other for infected sheep (Fig. 5a). However, in low-burden sheep there was no such clearly observable difference in infected and uninfected sheep (Fig. 5b). A similar trend was observed by PCoA based on the unweighted UniFrac distance (Fig. 5b). To determine which organisms differed in abundance before and after infection, sequence data were analysed at the phylum, family and genus level. The total number of phyla was similar in high-burden (24 phlya) and low-burden (26 phyla) sheep (Additional file 1: Figure S5). Significant differences in the relative abundances of four phyla were observed in high-burden sheep following infection, including the dominant phyla Firmicutes and Bacteroidetes. In low-burden sheep the relative abundances of two phyla differed significantly after infection, with both phyla being non-dominant (Fig. 6).

A total of 133 bacterial families were detected. In high-burden sheep, the abundance of 17 families differed significantly after infection including the dominant families Ruminococcaceae and Bacteroidaceae. In low-burden sheep the abundance of only seven families differed significantly after infection including Spirochaetaceae, S247 and Victivallaceae (Additional file 1: Figure S6).

**Fig. 7:**
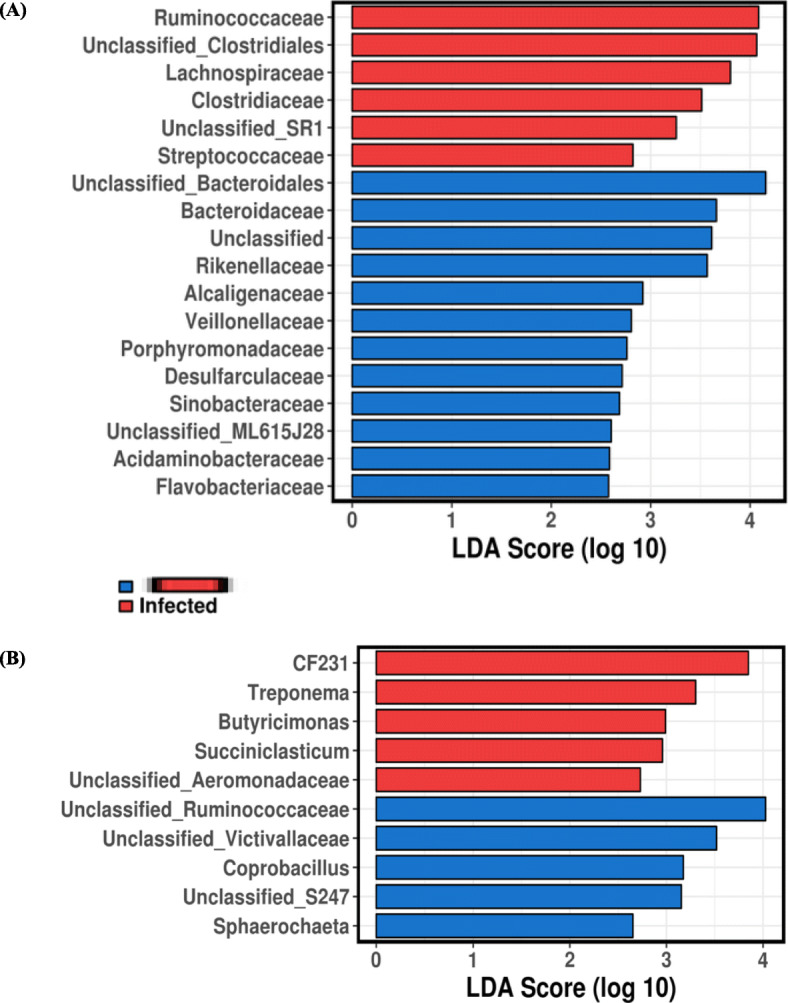
Significantly discriminative genera with absolute LDA score ≥ 2.0 between uninfected and infected group of sheep using LEfSe. (**a**) high-burden; (**b**) low-burden sheep

In high burden sheep, 207 genera were detected, with a similar number of genera (200) in low-burden sheep. In high-burden sheep, the relative abundance of 20 genera differed significantly following infection (Additional file 1: Table S5); whereas in low-burden sheep the relative abundance of only 10 genera differed significantly (Additional file 1: Table S6). There was no clear directional shift in abundance: some genera increased in abundance following infection, while others decreased in abundance. To further elucidate which genera contributed to the differences in microbial composition following infection, the relative abundances of genera were evaluated using LEfSe**.** In high-burden sheep 18 genera, and in low-burden sheep 10 genera, were determined to contribute to the differences in microbial composition following infection (Fig. 7).

Around 60% of the OTUs detected were present (in a minimum of one sample) both before and after *H. contortus* infection: this was the case for both high-burden and low-burden sheep (Additional file 1: Figure S7). When considering the core microbiota, 59% of OTUs were shared between uninfected and infected sheep for both high and low parasite burden groups (Additional file 1: Figure S8).

### The impact of *H. contortus* infection on microbial diversity

The Shannon, Simpson, Chao 1, richness, evenness and Fisher’s alpha diversity indices were used to compare microbial diversity in uninfected and infected sheep (Table 2). In low-burden sheep there was a trend of shift towards lower diversity of faecal microbiota following infection with *H. contortus* (*p* ≤ 0.06 for each diversity index used except the Chao1 index). In contrast, diversity appeared to increase following infection in high-burden sheep, though the differences were not significant (with the exception of Chao1 and Simpson diversity index).

**Table 2:** Microbial diversity indices of the faecal microbiota of uninfected and infected sheep

Diversity index	High-burden sheep			Low-burden sheep		
	Uninfected (mean ± SD)	Infected (mean ± SD)	p value	Uninfected (mean ± SD)	Infected (mean ± SD)	p value
Species richness	2335.50 ± 42.33	2402.30 ± 79.56	0.12	2383.0 ± 30.10	2181.67 ± 159.25	0.06
Species evenness	0.88 ± 0.01	0.89 ± 0.01	0.12	0.88 ± 0.01	0.86 ± 0.03	0.06
Chao1	3849.39 ± 101.84	4004.96 ± 197.77	0.06	3942.78 ± 74.1	3662.09 ± 227.41	0.12
Fisher’s alpha	956.86 ± 26.76	1003.67 ± 53.13	0.12	990.12 ± 18.94	863.44 ± 97.55	0.06
Shannon	6.83 ± 0.04	6.89 ± 0.08	0.12	6.86 ± 0.03	6.65 ± 0.26	0.06
Simpson	0.997 ± 0.001	0.998 ± 0.001	0.06	0.997 ± 0.001	0.995 ± 0.004	0.06

Significance determined using the Wilcoxon matched pairs signed rank test

## Discussion

This study identified associations between sheep faecal microbiota and *H. contortus* infection. Two key findings were of particular interest in this study. First, in the absence of infection (prior to experimental infection), sheep that go on to develop a high burden of infection have a faecal microbial composition that differs to sheep that subsequently develop a lower burden of infection. Secondly, following infection with *H. contortus*, sheep with a high-burden infection appear to undergo a greater change in their faecal microbial community structure than to sheep with a low-burden infection (relative to their respective pre-infection microbiota). These observations are based on the premise that fecundity (faecal egg counts) correlates with worm burden, as has been consistently shown in *H. contortus* infections [28–31].

Prior to infection with *H. contortus* there were several noticeable differences in the structure and composition of the faecal microbial communities of sheep that subsequently developed high burdens of infection relative to those that developed low burdens of infection. A clustering of high-burden sheep separate from low-burden sheep was observed for both ARISA and 16S rRNA data; indicative of differing microbial community structure. There are various aspects that impact upon community structure. In terms of the presence or absence of specific organisms, both ARISA and 16S rRNA sequencing revealed a large proportion (> 70% in ARISA and ~ 60% in 16S sequencing) of OTUs to be shared amongst high-burden and low-burden sheep. However, the relative abundance of important taxonomic groups differed in high-burden and low-burden sheep. Differences in abundance were observed in both dominant and sub-dominant taxa. Differences in microbiota were postulated following infection with the parasite, as have been shown for caprine infection with *H. contortus* [16], and are discussed below. However, such a segregation of host microbiota before infection in sheep that develop high-burden and low-burden infection after exposure to *H. contortus* is of particular interest. To our knowledge, such a comparative study has not been undertaken previously. The mechanisms behind this finding are currently unknown; but this preliminary work, if confirmed in larger cohorts, could have important ramifications in breed selection for parasite resistance and/or resilience.

Firmicutes and Bacteroidetes, which together make up ~ 80% of the total population in both the high- and low-burden groups, dominated the faecal microbiota of sheep in this study. These results are in line with the recent study of sheep faecal microbiota [10] and are supported by our recent findings [32]. However, there was a significant difference in relative abundance of Bacteroidetes (higher in high-burden sheep) and Firmicutes (lower in high-burden sheep) between high- and low-burden sheep. Both are known to be important (predominant) phyla in the gut microbiota of various animals, including cattle [8, 9], sheep [10], and humans [33, 34].

In humans, in which the vast majority of gut microbial composition studies have been conducted, both Bacteroidetes and Firmicutes are considered to be important phyla in healthy gut microbial communities. However, their exact role and the importance of their relative abundance has been debated. It has been suggested that the ratio of Firmicutes to Bacteroidetes may play a role in the development of obesity, with obese individuals having 20% more Firmicutes and almost 90% less Bacteroidetes compared to the lean individuals [35]. Moreover, higher Firmicutes-to-Bacteroidetes ratio was found to be strongly correlated with daily milk-fat yield in cows [36]. While obesity is obviously considered an undesirable state of health in humans, the propensity to gain weight (albeit muscle mass) is a desirable trait in animals reared for meat production. Here we observed higher Firmicutes-to-Bacteroidetes ratio in low-burden sheep (ratio 1.8:1) than high-burden (ratio 1.2:1) sheep (Additional file 1: Figure S9), which corresponds with the obese microbial composition in humans [35] and mice [37]. Though it should be noted that differences in proportions were considerably less in sheep than in obese humans. Nonetheless, it may be that the low-burden sheep have a microbial composition that favours their continued weight gain, and perhaps overall health. Interestingly, in the Australian sea lion, an animal with a thick layer of body fat for thermoregulation, there was a notable predominance of Firmicutes (80%) over Bacteriodes (2%) [38]. In contrast, De Filippo et al [39] found children in Africa had “significant enrichment in Bacteroidetes and depletion in Firmicutes” relative to European children. i.e. the high burden sheep had the same pattern as children that are more ‘susceptible’ (at least more exposed) to gastrointestinal pathogens. While the ratio of Firmicutes to Bacteroidetes is of interest, it is difficult to ascertain the importance of the relative abundance of two phyla, given the diversity of ecological and functional roles the many species within each phylum can play. It may be that the presence/absence of less dominant phyla, and the associated species/genera within those phyla, are equally or more important to gastrointestinal function and community structure than merely the ratio of the two dominant phyla.

Differences were detected in the relative abundance of subdominant phyla of bacteria. Significant differences were noted in the relative abundance of Verrucomicrobia, Tenericutes, Spirochaetes, Proteobacteria, Lentisphaerae, and Cyanobacteria. Proteobacteria are important as they include *Escherichia coli*, *Campylobacter*, and related Gram-negative bacilli. In humans it is often considered undesirable to have such bacteria in high numbers [40]; and here we see the Proteobacteria to be more abundant in high-burden sheep.

There were differences in relative abundances in some interesting genera. *Akkermansia* and *Dorea* were identified as the dominant groups in low burden sheep, whereas *Prevotella* was dominant in high burden sheep. The genus *Akkermansia* is of particular interest; a total of 50 different OTUs were associated with this genus (data not shown) with an average relative abundance of 1.13 and 2.39% in high and low parasite burden sheep, respectively. *Akkermansia* is a Gram-negative anaerobe in the human gut and has also been detected in the gastrointestinal tract of various other mammals [41]. *Akkermansia* uses mucin, a key component of mucous, as a source of energy. Thus, the bacterium is commonly associated with the mucous lining that covers the epithelial cells of much of the gastrointestinal tract. This mucous layer also acts as an adhesive surface for numerous microbes, facilitating host-microbe interactions. *A. muciniphila* colonises the intestine, protecting the gut from pathogens by means of competitive exclusion [42]. This bacterium colonises the human intestine at a very young age, possibly through the birthing process, or through feeding as it is found at low concentrations in breast milk and formula [43]. A low concentration of this species in human gut could indicate a thin mucous layer, thereby resulting in a weakened gut barrier function. Patients suffering from IBD, obesity and Type II diabetes tend to have lower concentrations of *A. muciniphila* [44]. Considering the development of third larval stage (L3) of *H. contortus* (which burrow into the gastric pits), and the key role mucous and associated molecules such as host galectins plays in resistance to infection [45], there may be a role for *Akkermansia* in maintenance of a healthy mucosa in sheep. Such modulation of mucin may hinder the development, establishment and feeding of the larval stage of *H. contortus*, by direct or indirect mechanisms. Such a scenario would ultimately impede the development of adult stage establishment and egg laying. The validity of such a hypothesis is yet to be established but warrants further consideration and investigation.

In addition to cluster analysis and difference in relative abundance of some taxa of interest, microbial diversity indices were also suggestive of a different community composition in high-burden and low-burden sheep prior to infection. Species richness, Chao1 and Fisher’s alpha tests were all suggestive of greater diversity in low-burden sheep. The exact role and/or importance of diversity in healthy gut function is unknown. In broad ecological terms, diversity is usually considered a desirable trait in natural ecosystems. Diversity has been assumed to be desirable in gut microbial communities too, notably in humans [46]. However, considering diversity alone, in the absence of species composition, is likely to be an overly simplistic measure of gut bacterial community health.

This study has demonstrated that infection of sheep with *H. contortus* clearly impacts upon the faecal microbial composition, and that the impact is greatest in high-burden sheep. To some degree, this finding is perhaps unsurprising, given the pathology that is likely to impact environmental conditions of the abomasum (e.g. pH change, presence of blood, mucosal damage) following infection [47]. However, what is of interest is the clear directional shift in community composition, and the extent of change in community composition in high-burden sheep.

In high-burden sheep a change in relative abundance in dominant taxa Firmicutes and Bacteroidetes was seen; with the shift going in the direction of what low-burden sheep have in the absence of infection (increase in Firmicutes and decrease in Bacteroidetes). However, though not significant, species richness was higher following infection in high burden sheep. Similarly, in goats species richness was observed to be higher in animals infected with *H. contortus* [16]. This might be due to the alterations in the composition of the major phyla like Firmicutes and Bacteroidetes, and subsequently, introduction of less dominant species to fill the vacated niches. In contrast, the only significant difference in abundance of phyla in low-burden sheep was in sub-dominant phyla, suggesting a lesser overall impact on community composition. ANOSIM based on weighted and unweighted UniFrac suggested that number of OTUs rather than the abundance of OTUs played a major role in the differences between infected and uninfected groups of sheep. This premise is supported to some extent by diversity indices. For all indices tested, there was a trend (*p* ≤ 0.06) towards lesser species diversity in low-burden sheep following infection. It could be hypothesized that the presence of less adult parasites in low-burden sheep made less physiological changes in the gut, conferring a reduced alteration of community composition. Conversely, a high burden of adult parasites could result in large physiological changes in the gut; conferring increased alteration of community composition.

Throughout this study, we used faecal pellets for analysis on the assumption that bacteria in faeces represent the microbial community of the digestive tract. However, the use of faecal samples in a study such as this does have some limitations. The microbiota of the different compartments of the ruminant gut are different, and it is more distinct between upper and lower digestive tract [48]. The faecal microbiota of the leopard seal was more similar to the microbiota from sections of the large intestine than the small intestine [49], and this is likely to hold true for all higher animals: microbial composition of faeces is likely to most closely resemble that of the lower digestive tract. *H. contortus* infects the abomasum, thus the microbial community structure of that section of the gastrointestinal tract may be of most relevance when investigating susceptibility or resistance to *H. contortus* infection. However, compartments of the digestive tract are intimately connected via various physiological body systems. Hence, changes in one compartment are likely to cause physiological and immunological changes in other areas of the gastrointestinal tract [50]. Such changes would be expected to cause perturbations in the respective microbial communities. Due to this interconnectivity, as well as the linear physical nature of the digestive tract, where microbes present in the upper digestive tract exit the body through the lower digestive tract, faeces is a viable specimen for these studies.

## Conclusions

This study has highlighted an area of research which is currently lacking. The findings demonstrated that parasite infection clearly altered the gut microbiota of infected sheep. The initial work presented here also suggests that microbiota may vary between sheep with differing disease susceptibility/resistance to the most globally significant nematode parasite of small ruminants. The implication of this finding for broader gastrointestinal parasite resistance is difficult to ascertain at this stage. Further studies are clearly warranted and suggest parasite-microbe interactions may have important impacts on productivity in small ruminants.

## Materials and methods

### Experimental animals and sampling

The 28 Merino wethers (2-year-old) used in this experiment were derived from the Sheep CRC Information Nucleus Flock (http://www.sheepcrc.org.au/). All sheep were considered to have a wide spectrum of parasite resistance, as measured by their Australian Sheep Breeding Value.

After arrival to the Animal Facility, all sheep were treated with anthelmintic (Cydectin®) once, and each animal was confirmed as uninfected by faecal egg count (FEC) prior to experimental infection. The animals were kept indoors on raised flooring and fed ad libitum, and housed together ~ 6 months prior to commencement of the experimental sample collection.

Each animal was infected with two doses of 7000 *H. contortus* L3 larvae given 3 days apart (14,000 larvae in total). At 21 days post-infection, FEC were performed twice a week for 3 weeks (168 samples) using the McMaster method to determine the burden of infection in 28 sheep. After six counts, the cumulative FEC (cFEC) was taken to determine the status of infection.

**Fig. 8:**
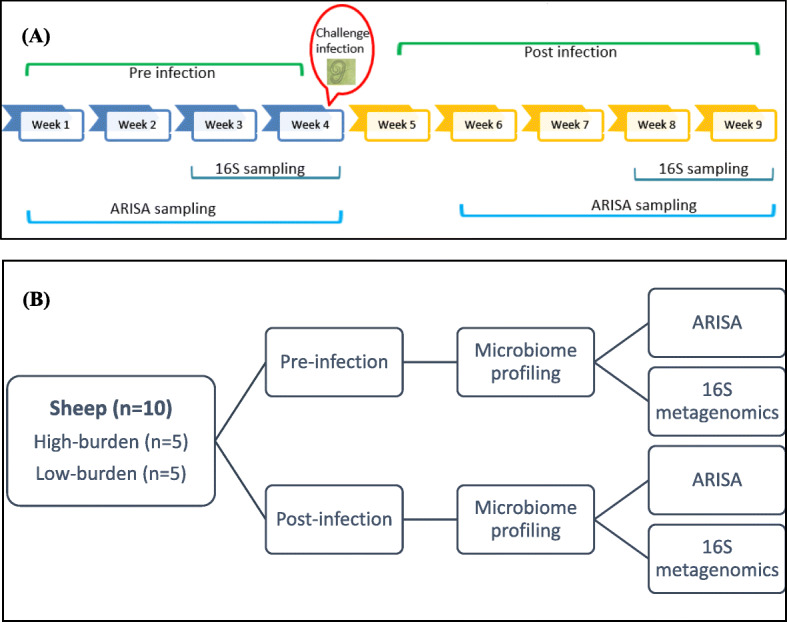
Diagrammatic representation of experimental design. (**a**): Time course of sampling and experimental infection; (**b**): Analytical procedure for sheep before and after experimental infection with *H. contortus*

Faecal samples (*n* = 28) were collected for 8 of the 9 weeks of the study; 4 weeks before and 4 weeks after infection (Fig. 8) (a total of 224 sample collected). Samples were collected directly from the rectum of sheep by hand covered with sterile gloves. Immediately after collection, samples were taken to the laboratory in an ice box (‘esky’), labelled and stored at − 80 °C. Out of 28 sheep sampled, faecal samples from the five highest-burden (with highest egg count) and five lowest-burden (with lowest egg count) sheep (as determined by the cFEC) were used for subsequent bacterial community profiling.

### Faecal bacteria community profiling

Archived faecal samples from 10 selected sheep (five high-burden and five low-burden) were analysed by ARISA and 16S rRNA gene sequencing. Samples collected over eight sampling events: four prior to infection and four after infection (80 samples) were analysed by ARISA. Details of data analysis for ARISA were discussed in our previous manuscript [32], and also provided in the supplementary material (Additional file 1).

For 16S rRNA gene sequencing, 40 samples were analysed, consisting of samples from the 2 weeks immediately prior to infection, and for 2 weeks when adult worms were likely to be present (i.e. 3–4 weeks post infection).

Genomic DNA was extracted from frozen faecal samples using the QIAamp DNA Stool Kit reagents with the EconoSpin column (Epoch Life Science, Inc., Missouri City, USA) following the QIAamp DNA Stool Kit protocol. Extracted DNA was quantified using Qubit® Fluorometer according to the manufacturer’s instructions.

### 16S rRNA gene sequencing

16S rRNA gene sequencing was conducted to characterise community profiles and identify important genera. PCR targeting the V1-V3 region of the 16S rRNA gene was conducted using primers 27F (AGAGTTTGATCMTGGCTCAG) and 519R (GWATTACCGCGGCKGCTG) [18, 51]. Amplicon sequencing was performed on the MiSeq platform utilising Illumina’s paired end chemistry. All 40 samples that had 16S sequencing conducted were included in the same sequencing run. Paired-end reads were assembled by merging the forward and reverse reads using PEAR (version 0.9.5) [52]. Primers were trimmed using Seqtk (version 1.0) [53], then sequences were quality filtered with a maximum expected error threshold of 0.5, full length duplicate sequences were removed, and sequences sorted by abundance using USEARCH [54, 55]. Singletons or unique reads in the data set were discarded. Sequences were clustered using USEARCH at 97% similarity, and subsequently chimera filtered using “rdp_gold” database as reference. To obtain the number of reads of each OTU, reads were mapped back to OTUs with a minimum identity of 97%. Taxonomy was assigned using Greengenes database [56] (Version 13_8, Aug 2013) by QIIME [57]. A rarefied (10,000 sequences per sample) biom table was imported to Calypso [58] for further analyses.

### Ecological and statistical analyses

ARISA abundance data and 16S square root transformed abundance data were used to generate Bray-Curtis similarities [59], and weighted and unweighted UniFrac distance matrices. Similarities between sample groups were visualised using non-metric multi-dimensional scaling (nMDS) [60], and principal coordinates analysis (PCoA) plot. To test for differences in composition of the faecal microbiota between sheep and over time, Analysis of Similarity (ANOSIM) was performed. ANOSIM produces a statistic, R, taking a value usually between 0 and + 1. The statistical significance of R under the null hypothesis of no separation among groups was also tested [32]. Significant differences (cut-off: *p* ≤ 0.05) in egg counts, microbial diversity and abundances were tested using t-test, Mann Whitney test and Wilcoxon matched-pairs signed rank test in GraphPad Prism version 6.0. All other statistical tests and plotting for ARISA data were performed using the software PRIMER-E v7 [61]. Calypso was used to analyse and plot 16S rRNA gene sequencing data. The differences in faecal microbial composition (relative abundance) between high-burden and low-burden sheep, prior and following infection was compared using Linear Discriminant Analysis (LDA) Effect Size (LEfSe) algorithm [62] in Calypso.

## Supplementary information

**Additional file 1.** **ARISA results.** *Community Structure of Faecal Microbiota of High- and Low-Burden Sheep Prior to Infection.*A three-way ANOSIM of ARISA data revealed a separation (*R* = 0.243, *p* ≤ 0.01) of microbial composition between high- and low-burden sheep. Differences in the microbial composition were visualised by nMDS plot, showing distinct clustering of faecal microbiota in high-burden sheep relative to low-burden sheep (Additional file 1: Figure S10). Most OTUs were shared among high and low parasite burden sheep groups (Additional file 1: Figure S11), but abundances of dominant OTUs differed (Additional file 1: Figure S12). *Community Structure of Faecal Microbiota of High- and Low-Burden Sheep Before and After H. contortus Infection*. Three-way ANOSIM of ARISA data revealed separation (*R* = 0.243, *p* ≤ 0.01) of gut microbial composition following H. contortus infection in high-burden sheep, and no clear separation in low-burden sheep (*R* = 0.190, *p* ≤ 0.01). An nMDS plot showed a lack of distinct clustering of gut microbiota in uninfected and infected sheep with high worm burden, although there was a predominance of infected sheep samples in the upper left section of the plot (Additional file 1: Figure S13a). No clear clustering was observed in low-burden sheep (Additional file 1: Figure S13b). There were no significant differences in the relative abundances of the dominant OTUs following infection (Figure S14). **ARISA data analysis.** ARISA was used to determine community profiles. Quantified DNA was diluted to 20 ng/μL using RNase free water for use as template for PCR [63, 64]. PCR amplification of the ITS region was performed in duplicate using the previously described primer set ITSF/ITSReub [65–68] with HotStarTaq® Plus master mix. Fragment separation was conducted using an Applied Biosystems 3730 DNA analyser with a GS1200 LIZ® internal size standard. Peak size, height and area data were extracted to Microsoft Excel after performing accurate size calling by using GeneMapper software Version 4.0 for further analysis. The software converted fluorescence data into electropherograms; peaks represented fragments of different sizes, and the peak’s areas represented the relative proportion of the fragments. All peaks with fluorescent intensity of ≤50 relative fluorescence units were excluded as they might be the part of instrumental noise (sometimes referred to as background peaks) [64, 66, 69–71]. Given the approximate minimal known lengths of the ITS region (143 bp) [70] included in the primer sets ITSC and 1552/132, fragment lengths below 229 bp and 300 bp, respectively, were eliminated from analysis. Data comprising the true peak sizes and peak areas were converted to abundance per binned operational taxonomic units (OTUs) using the custom binning script interactive binner [64] in the R software package [72], with a relative fluorescence intensity cut-off of 0.09%, a window size (WS) of two and a shift size of 0.1 [64]. To determine the best binning strategy for a dataset without a priori knowing the ideal WS value, the script automatic binner [64] in R was used which allows for an automatic calculation of a series of WS values (e.g. 0.5, 1, 2, 3, 4, and 5 bp) for a given shift value (e.g. 0.1 bp). A compromise between high resolution (low WS) and high similarity among samples (high WS) was made based on the output of the script. **Figure S1** Rarefaction curve based on OTU richness values. **Figure S2** The bacterial families with significant differences in relative abundances in high and low burden sheep (total family = 133). The Y-axis shows the average relative abundances (%); X-axis shows family. Unpaired t-test. Error bars represent SD. *p ≤ 0.05, **p ≤ 0.01, ***p ≤ 0.001. **Figure S3** Venn diagram representing the shared and unique OTUs in sheep with high and low burdens of parasite. A bacterial group was considered to be present in a sample group if it was identified in at least 10% of the samples within the group. **Figure S4** Venn diagram representing the core OTUs in group of sheep with high and low burden of parasite. A bacterial group was considered to be present in a sample group if it was identified in at least 50% of the samples within the group. **Figure S5** HeatMap + of the relative abundances of the identified phyla in uninfected and infected sheep. (**a**) high-burden; (**b**) low-burden sheep. The maps showed marked differences in relative abundances in high-burden sheep compared to low-burden sheep. **Figure S6** The bacterial families with significant differences in relative abundances before and after infection. (**a**) high-burden; (**b**) low-burden sheep. The Y-axis shows the average relative abundances (%); X-axis shows family. Pair-wise comparisons are done by unpaired t-test. Error bars represent SD. *p ≤ 0.05, **p ≤ 0.01, ***p ≤ 0.001. **Figure S7** Venn diagram representing the shared and unique OTUs present in sheep before and after infection. (**a**) high-burden sheep; (**b**) low-burden sheep. A bacterial group was marked as present in a sample group if it was identified in at least 10% of the samples within the group. **Figure S8** Venn diagram representing the core OTUs present in sheep before and after infection. (**a**) high-burden sheep; (**b**) low-burden sheep. An OTU was considered as core if it was identified in at least 50% of the samples within the group. **Figure S9** The two most dominant bacterial phyla with significant differences in relative abundances between high- and low burden sheep prior to infection. The Y-axis shows the average relative abundances (%); X-axis shows phyla. Significance determined using Mann Whitney test. Error bars represent SD. ****p ≤ 0.0001. **Figure S10** Distinct clustering of gut microbiome between high and low parasite burden sheep (before infection) using ARISA data. nMDS plot based on Bray-Curtis similarity matrix of gut bacterial composition (10 sheep, 4 sampling events). Each symbol represents an individual sheep sample at a given time. Blue indicates high-burden and red represents low-burden sheep. **Figure S11** Venn diagram representing the shared and unique OTUs in high and low parasite burden sheep as determined by ARISA sampled over 4 weeks. An OTU was considered to be present in a sample group if it was identified in at least one of the samples within the group. **Figure S12** Average relative abundances of 10 most dominant OTUs of high-burden and low-burden sheep, as determined by ARISA. The Y-axis shows the average relative abundances (%). X-axis represents OTUs. Unpaired t- test. Error bars represent SD. *p ≤ 0.05, **p ≤ 0.01, ***p ≤ 0.001. **Figure S13** Clustering of gut microbiome of uninfected and infected sheep using ARISA data. (**a**) high-burden; (**b**) low-burden sheep. nMDS plot of sheep based on Bray-Curtis similarity matrix of faecal microbial composition. Blue indicates uninfected whereas red represents infected sheep sample. Sampling were conducted over eight weeks, giving rise to 40 dot points for both high and low burden sheep. **Figure S14** Average relative abundances of commonly detected OTUs of uninfected and infected sheep detected by ARISA. (**a**) high-burden; (**b**) low-burden sheep. The Y-axis shows the average relative abundances (%). X-axis represents OTUs. Error bars represent SD. (Paired t-test; no significance was observed). **Table S1** ANOSIM of microbial composition of sheep between high-burden and low-burden sheep (16S data)**.** **p ≤ 0.01**.** **Table S2** Identified genera with significantly different abundances between the high and low burden sheep (16S data). Significance determined using the Wilcoxon matched pairs signed rank test. *p ≤ 0.05, **p ≤ 0.01, ***p ≤ 0.001, ****p ≤ 0.0001. **Table S3** The 50 most abundant core OTUs with different abundances between the high and low parasite burden sheep (16S data). **Table S4** ANOSIM of microbial composition of high and low parasite burden sheep following infection (16S data). **p ≤ 0.01, ***p ≤ 0.001**. Table S5** The genera with significantly different abundances between the uninfected and infected sheep with high parasite burden (*n* = 20) (16S data). Significance determined using the Wilcoxon matched pairs signed rank test. *p ≤ 0.05, **p ≤ 0.01, ***p ≤ 0.001, ****p ≤ 0.0001**. Table S6** The genera with significantly different abundances between the uninfected and infected sheep with low parasite burden (*n* = 20) (16S data). Significance determined using the Wilcoxon matched pairs signed rank test. *p ≤ 0.05, **p ≤ 0.01

## Data Availability

The 16S rRNA gene sequence data were deposited in the NCBI Sequence Read Archive (SRA) under the project accession number - PRJNA561335.

## Abbreviations

ANOSIM: Analysis of similarity
ANOVA: Analysis of variance
ARISA: Automated ribosomal intergenic spacer analysis
cEPG: Cumulative egg per gram of faeces
cFEC: Cumulative faecal egg count
IBD: Inflammatory bowel disease
ITS: Internal transcribed spacer
LEfSe: Linear discriminant analysis effect size
nMDS: Non-metric multidimensional scaling
OTU: Operational taxonomic unit
PCoA: Principal coordinate analysis
PCR: Polymerase chain reaction
QIIME: Quantitative Insights Into Microbial Ecology
rRNA: Ribosomal ribonucleic acid
rRNA: Ribosomal ribonucleic acid
SD: Standard deviation
WS: Window size
