# Supplementary material for: Variation in gut bacterial composition is associated with Haemonchus contortus parasite infection of sheep
Source: Anim Microbiome. 2020 Feb 5;2:3. doi: 10.1186/s42523-020-0021-3 (PMC7807447; doi:10.1186/s42523-020-0021-3)
Supplement: Supplementary file 1 — Additional file 1. ARISA results. Community Structure of Faecal Microbiota of High- and Low-Burden Sheep Prior to Infection.A three-way ANOSIM of ARISA data revealed a separation (R = 0.243, p ≤ 0.01) of microbial composition between high- and low-burden sheep. Differences in the microbial composition were visualised by nMDS plot, showing distinct clustering of faecal microbiota in high-burden sheep relative to low-burden sheep (Additional file 1: Figure S10). Most OTUs were shared among high and low parasite burden sheep groups (Additional file 1: Figure S11), but abundances of dominant OTUs differed (Additional file 1: Figure S12). Community Structure of Faecal Microbiota of High- and Low-Burden Sheep Before and After H. contortus Infection. Three-way ANOSIM of ARISA data revealed separation (R = 0.243, p ≤ 0.01) of gut microbial composition following H. contortus infection in high-burden sheep, and no clear separation in low-burden sheep (R = 0.190, p ≤ 0.01). An nMDS plot showed a lack of distinct clustering of gut microbiota in uninfected and infected sheep with high worm burden, although there was a predominance of infected sheep samples in the upper left section of the plot (Additional file 1: Figure S13a). No clear clustering was observed in low-burden sheep (Additional file 1: Figure S13b). There were no significant differences in the relative abundances of the dominant OTUs following infection (Figure S14). ARISA data analysis. ARISA was used to determine community profiles. Quantified DNA was diluted to 20 ng/μL using RNase free water for use as template for PCR [63, 64]. PCR amplification of the ITS region was performed in duplicate using the previously described primer set ITSF/ITSReub [65–68] with HotStarTaq® Plus master mix. Fragment separation was conducted using an Applied Biosystems 3730 DNA analyser with a GS1200 LIZ® internal size standard. Peak size, height and area data were extracted to Microsoft Excel after performing accurate size calling by [file 42523_2020_21_MOESM1_ESM.pdf]

Additional file 1

ARISA results

Community Structure of Faecal Microbiota of High- and Low-Burden Sheep Prior to Infection

A three-way ANOSIM of ARISA data revealed a separation ($R = 0.243$, $p \leq 0.01$) of microbial composition between high- and low-burden sheep. Differences in the microbial composition were visualised by nMDS plot, showing distinct clustering of faecal microbiota in high-burden sheep relative to low-burden sheep (Additional file 1: Figure S10). Most OTUs were shared among high and low parasite burden sheep groups (Additional file 1: Figure S11), but abundances of dominant OTUs differed (Additional file 1: Figure S12).

Community Structure of Faecal Microbiota of High- and Low-Burden Sheep Before and After *H. contortus* Infection

Three-way ANOSIM of ARISA data revealed separation ($R = 0.243$, $p \leq 0.01$) of gut microbial composition following *H. contortus* infection in high-burden sheep, and no clear separation in low-burden sheep ($R = 0.190$, $p \leq 0.01$). An nMDS plot showed a lack of distinct clustering of gut microbiota in uninfected and infected sheep with high worm burden, although there was a predominance of infected sheep samples in the upper left section of the plot (Additional file 1: Figure S13a). No clear clustering was observed in low-burden sheep (Additional file 1: Figure S13b). There were no significant differences in the relative abundances of the dominant OTUs following infection (Figure S14).

ARISA data analysis

ARISA was used to determine community profiles. Quantified DNA was diluted to 20ng/µL using RNase free water for use as template for PCR [63, 64]. PCR amplification of the ITS region was performed in duplicate using the previously described primer set ITSF/ITSReub [65-68] with HotStarTaq® Plus master mix. Fragment separation was conducted using an Applied Biosystems 3730 DNA analyser with a GS1200 LIZ ® internal size standard.

Peak size, height and area data were extracted to Microsoft Excel after performing accurate size calling by using GeneMapper software Version 4.0 for further analysis. The software converted fluorescence data into electropherograms; peaks represented fragments of different sizes, and the peak's areas represented the relative proportion of the fragments. All peaks with fluorescent intensity of ≤50 relative fluorescence units were excluded as they might be the part of instrumental noise (sometimes referred to as background peaks) [64, 66, 69-71]. Given the approximate minimal known lengths of the ITS region (143bp) [70] included in the primer sets ITSC and 1552/132, fragment lengths below 229bp and 300bp, respectively, were eliminated from analysis. Data comprising the true peak sizes and peak areas were converted to abundance per binned operational taxonomic units (OTUs) using the custom binning script interactive binner [64] in the R software package [72], with a relative fluorescence intensity cut-off of 0.09%, a window size (WS) of two and a shift size of 0.1 [64]. To determine the best binning strategy for a dataset without a priori knowing the ideal WS value, the script automatic binner [64] in R was used which allows for an automatic calculation of a series of WS values (e.g. 0.5, 1, 2, 3, 4, and 5 bp) for a given shift value (e.g. 0.1 bp). A compromise between high resolution (low WS) and high similarity among samples (high WS) was made based on the output of the script.

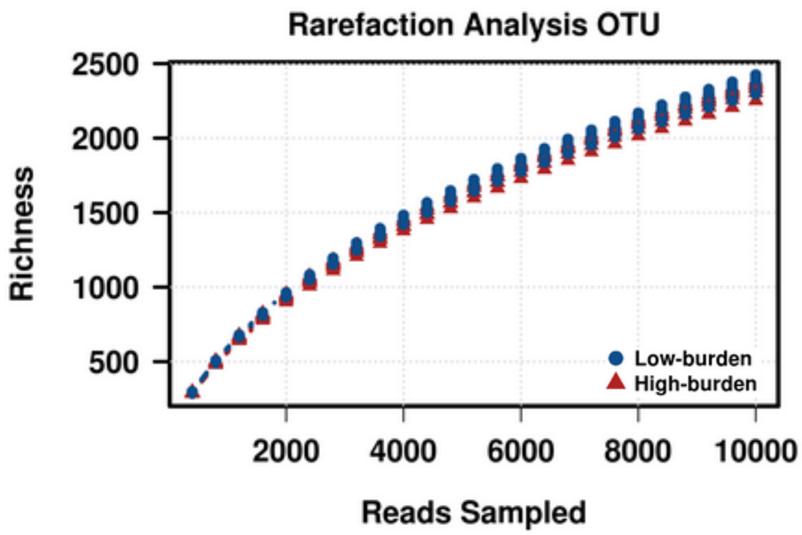

Figure S1 Rarefaction curve based on OTU richness values.

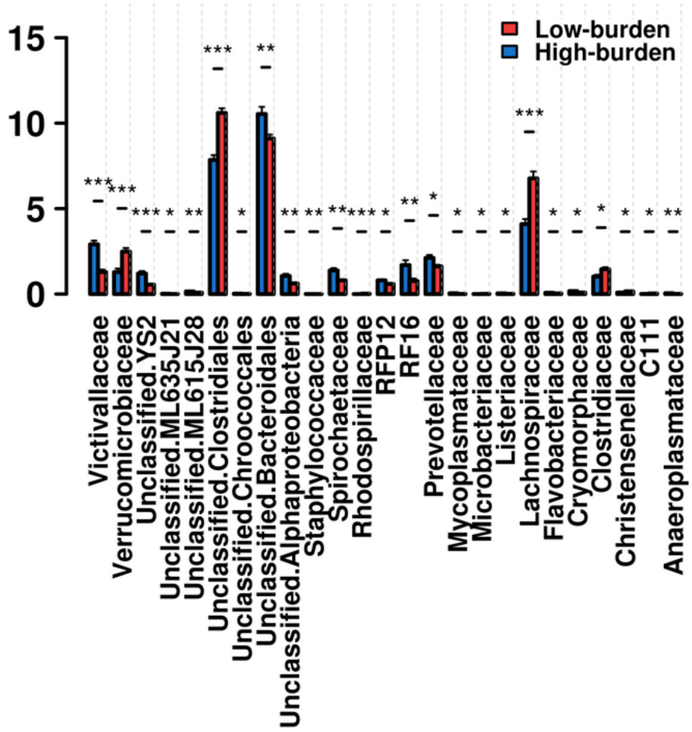

Figure S2 The bacterial families with significant differences in relative abundances in high and low burden sheep (total family = 133). The Y-axis shows the average relative abundances (%); X-axis shows family. Unpaired t-test. Error bars represent SD. *p ≤ 0.05, **p ≤ 0.01, ***p ≤ 0.001.

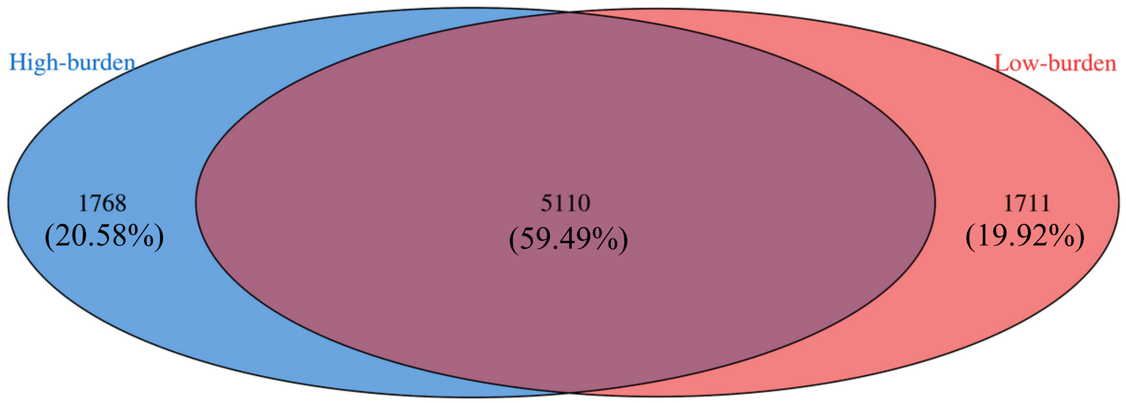

Figure S3 Venn diagram representing the shared and unique OTUs in sheep with high and low burdens of parasite. A bacterial group was considered to be present in a sample group if it was identified in at least 10% of the samples within the group.

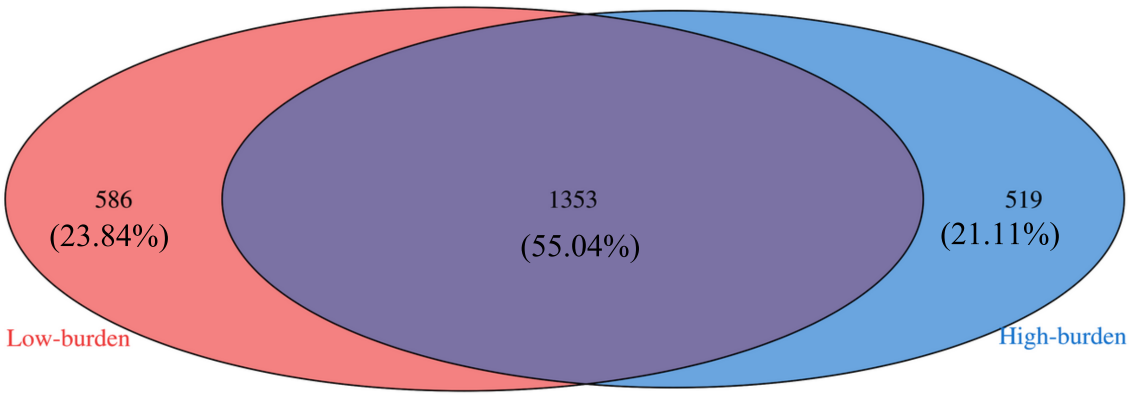

Figure S4 Venn diagram representing the core OTUs in group of sheep with high and low burden of parasite. A bacterial group was considered to be present in a sample group if it was identified in at least 50% of the samples within the group.

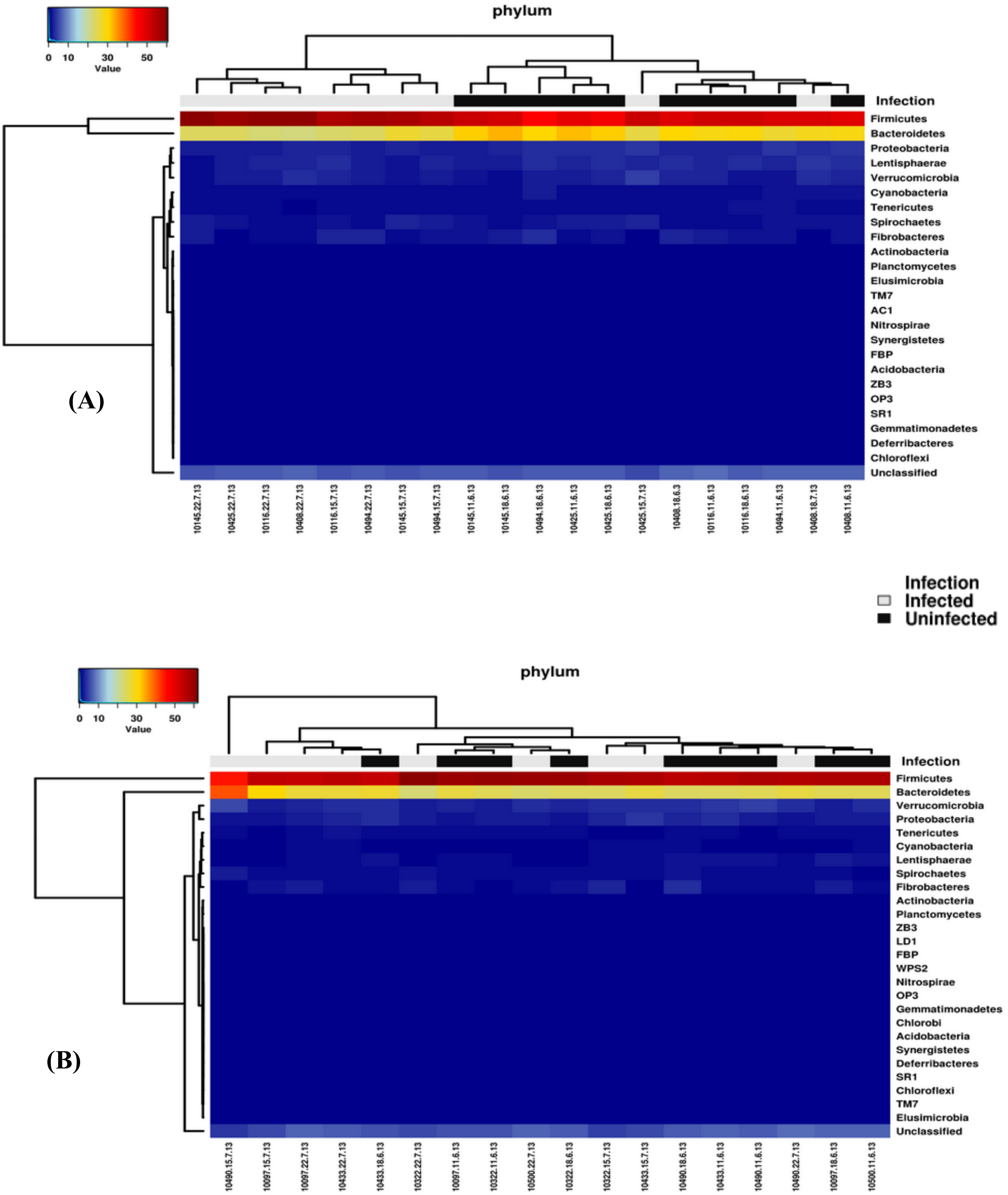

Figure S5 HeatMap+ of the relative abundances of the identified phyla in uninfected and infected sheep. (**a**) high-burden; (**b**) low-burden sheep. The maps showed marked differences in relative abundances in high-burden sheep compared to low-burden sheep.

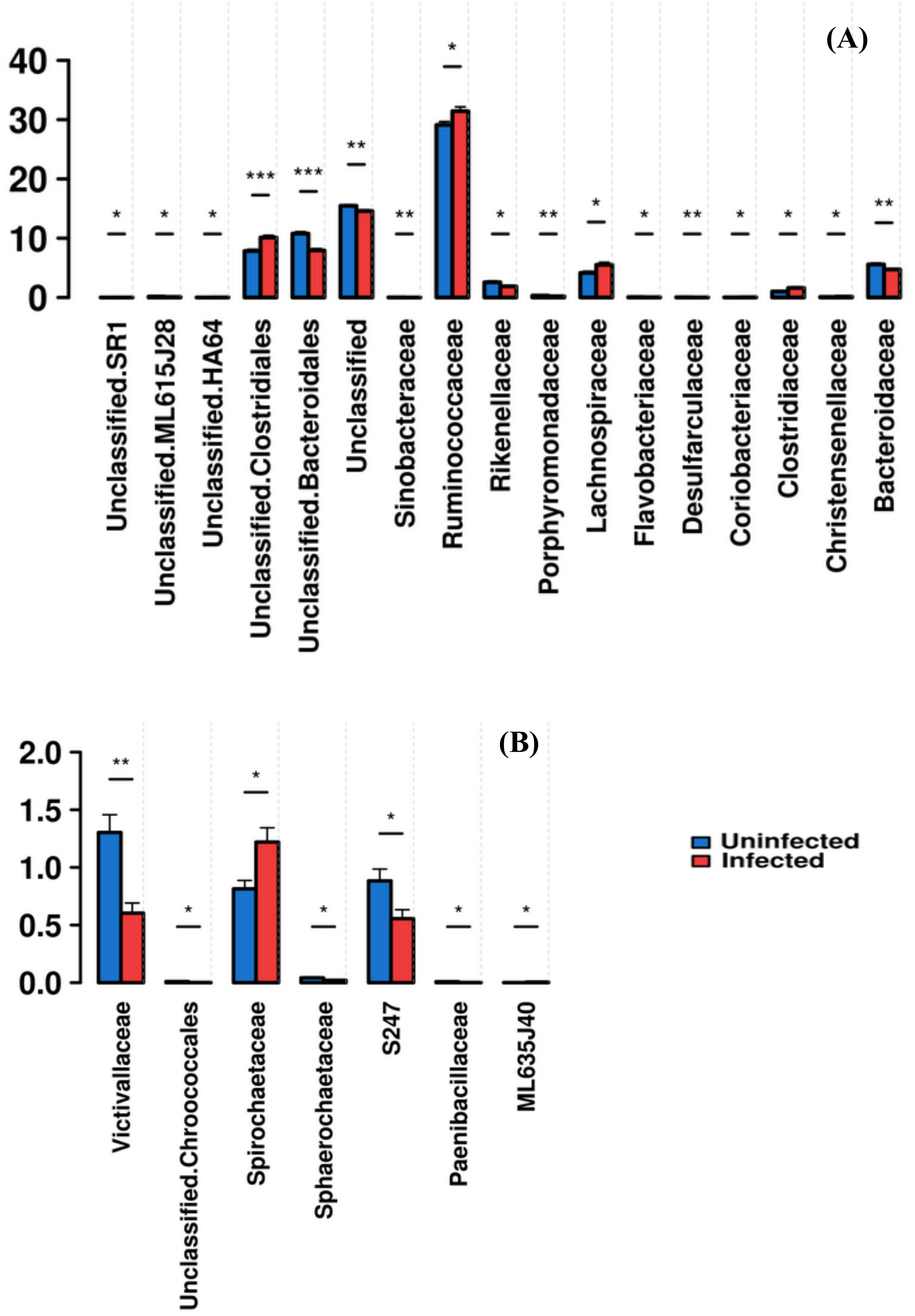

Figure S6 The bacterial families with significant differences in relative abundances before and after infection. (**a**) high-burden; (**b**) low-burden sheep. The Y-axis shows the average relative abundances (%); X-axis shows family. Pair-wise comparisons are done by unpaired t-test. Error bars represent SD. $^*p \le 0.05$, $^{**}p \le 0.01$, $^{***}p \le 0.001$.

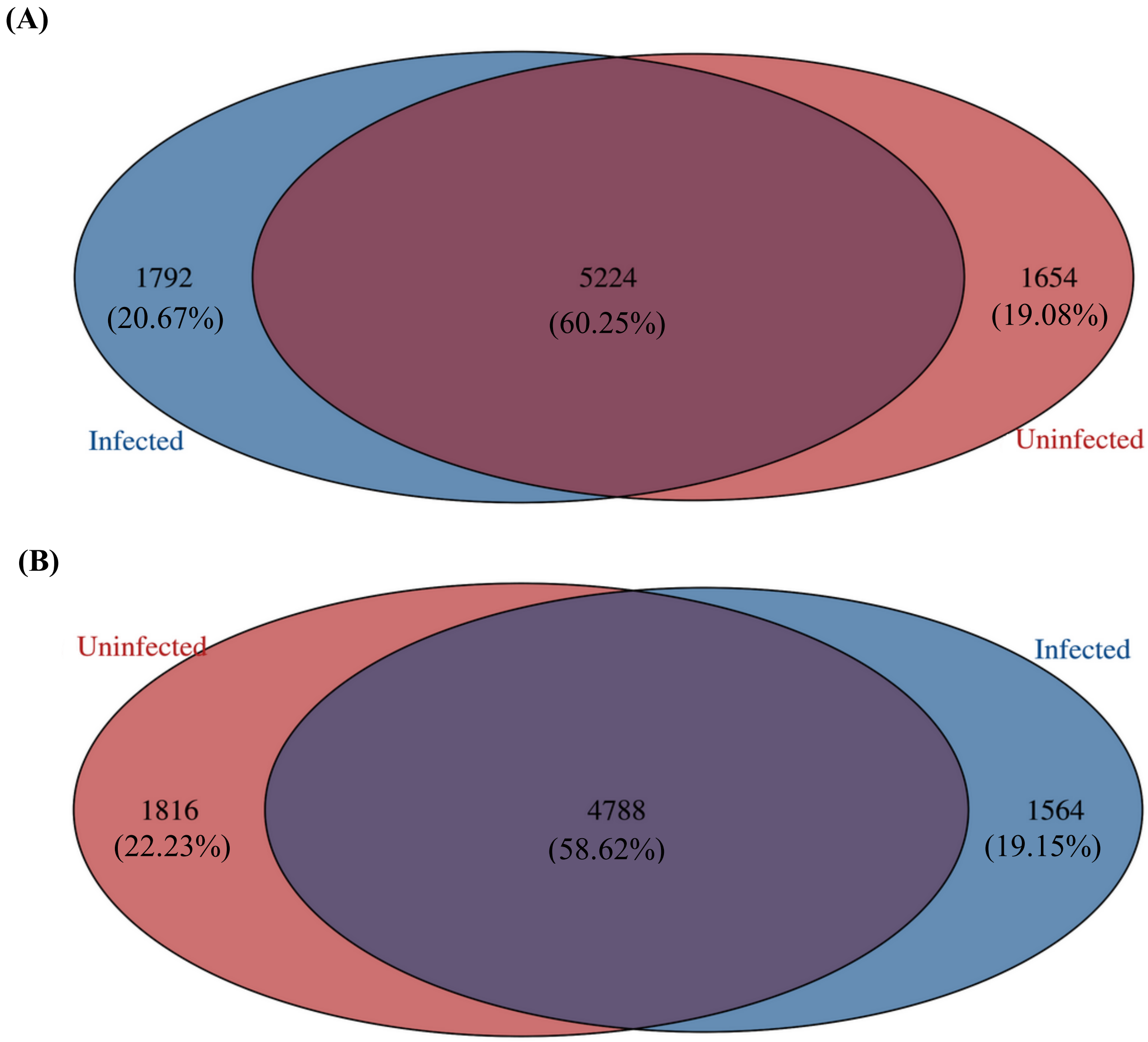

Figure S7 Venn diagram representing the shared and unique OTUs present in sheep before and after infection. (**a**) high-burden sheep; (**b**) low-burden sheep. A bacterial group was marked as present in a sample group if it was identified in at least 10% of the samples within the group.

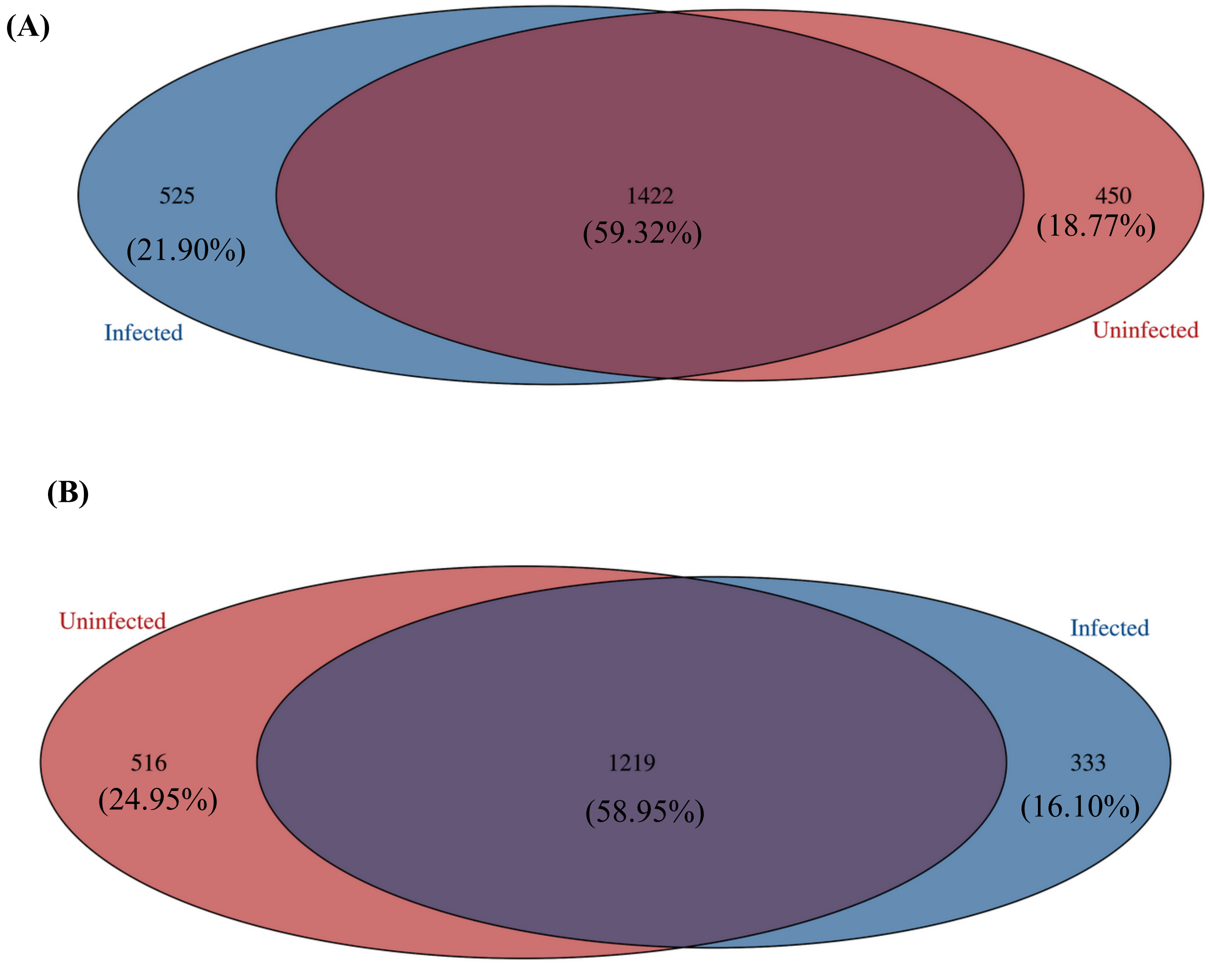

Figure S8 Venn diagram representing the core OTUs present in sheep before and after infection. (**a**) high-burden sheep; (**b**) low-burden sheep. An OTU was considered as core if it was identified in at least 50% of the samples within the group.

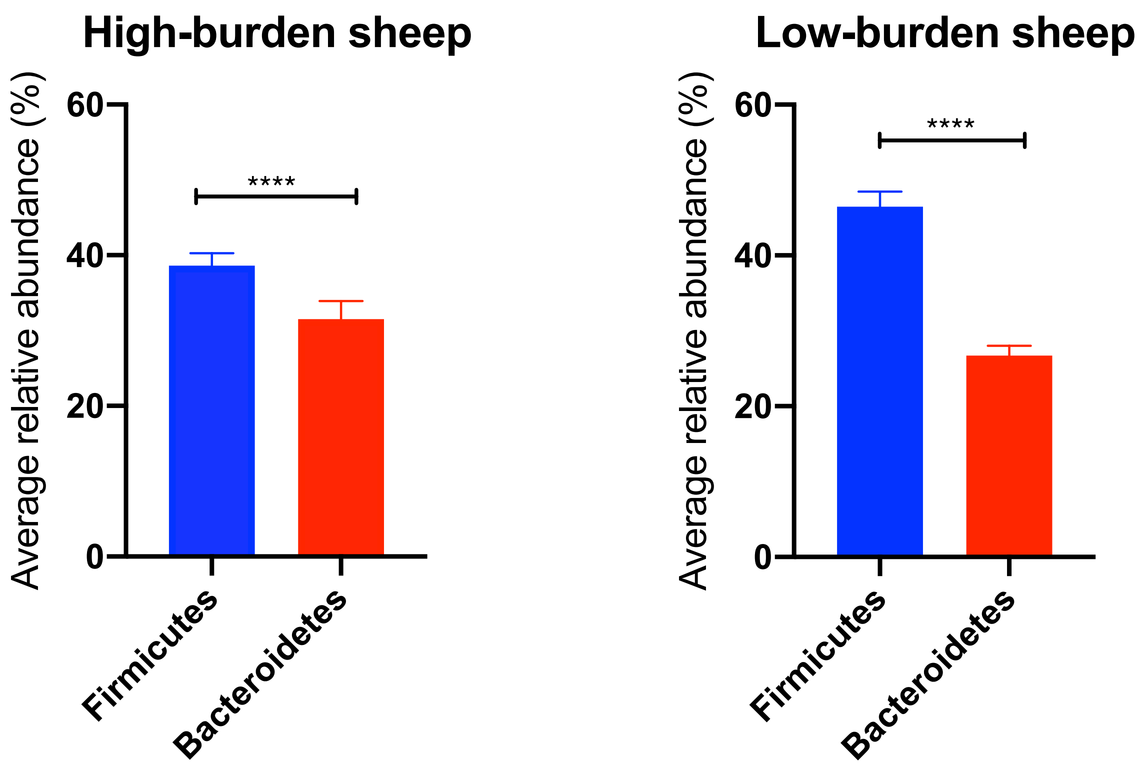

Figure S9 The two most dominant bacterial phyla with significant differences in relative abundances between high- and low burden sheep prior infection. The Y-axis shows the average relative abundances (%); X-axis shows phyla. Significance determined using Mann Whitney test. Error bars represent SD. ****$p \leq 0.0001$.

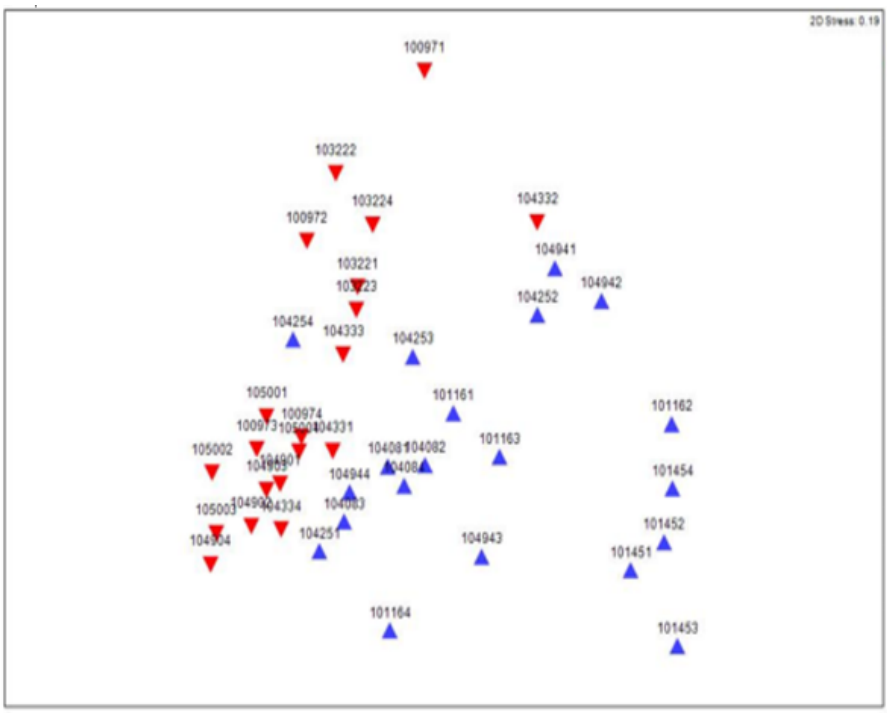

Figure S10 Distinct clustering of gut microbiome between high and low parasite burden sheep (before infection) using ARISA data. nMDS plot based on Bray-Curtis similarity matrix of gut bacterial composition (10 sheep, 4 sampling events). Each symbol represents an individual sheep sample at a given time. Blue indicates high-burden and red represents low-burden sheep.

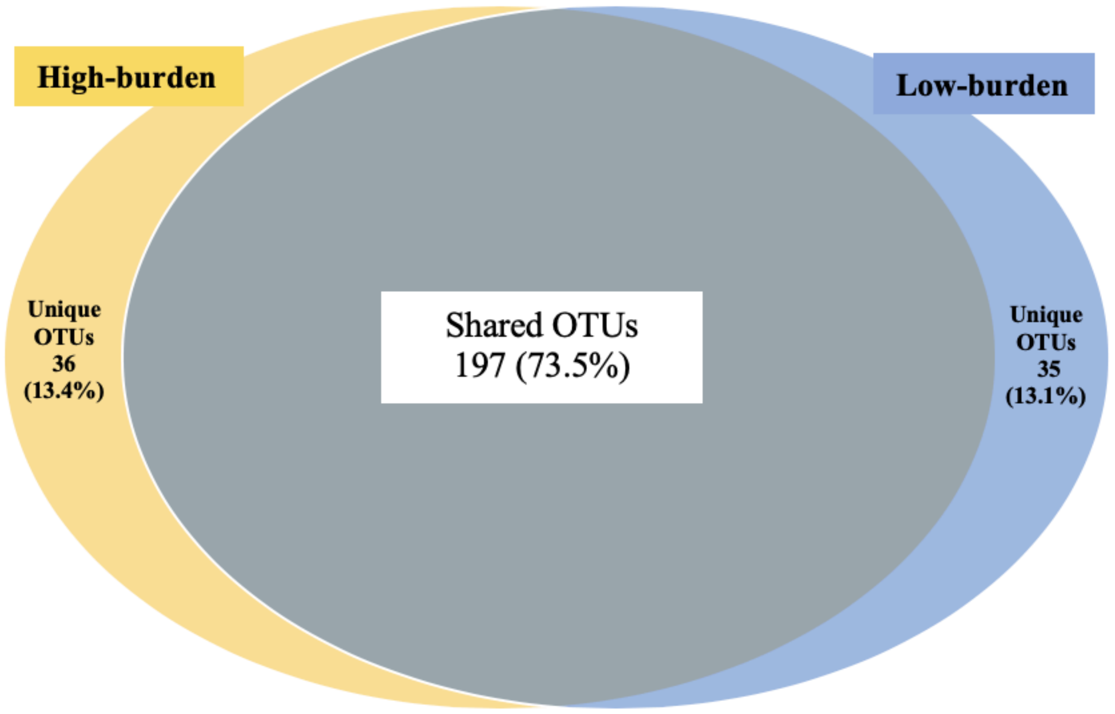

Figure S11 Venn diagram representing the shared and unique OTUs in high and low parasite burden sheep as determined by ARISA sampled over 4 weeks. An OTU was considered to be present in a sample group if it was identified in at least one of the samples within the group.

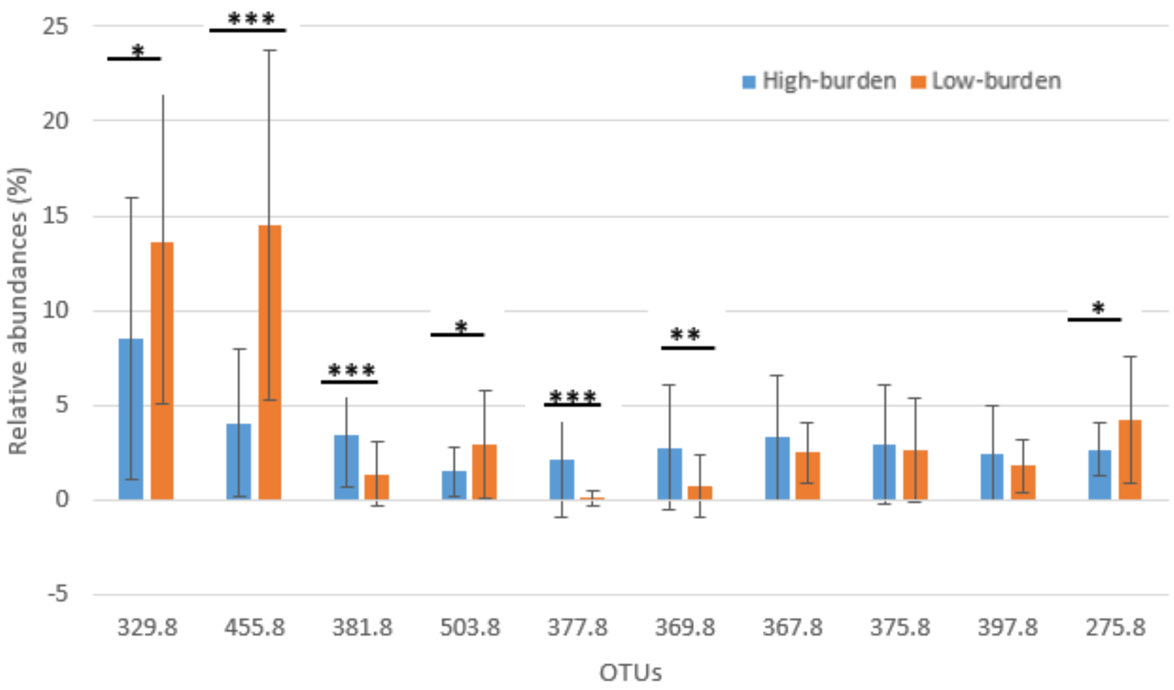

Figure S12 Average relative abundances of 10 most dominant OTUs of high-burden and low-burden sheep, as determined by ARISA. The Y-axis shows the average relative abundances (%). X-axis represents OTUs. Unpaired t- test. Error bars represent SD. *p ≤ 0.05, **p ≤ 0.01, ***p ≤ 0.001.

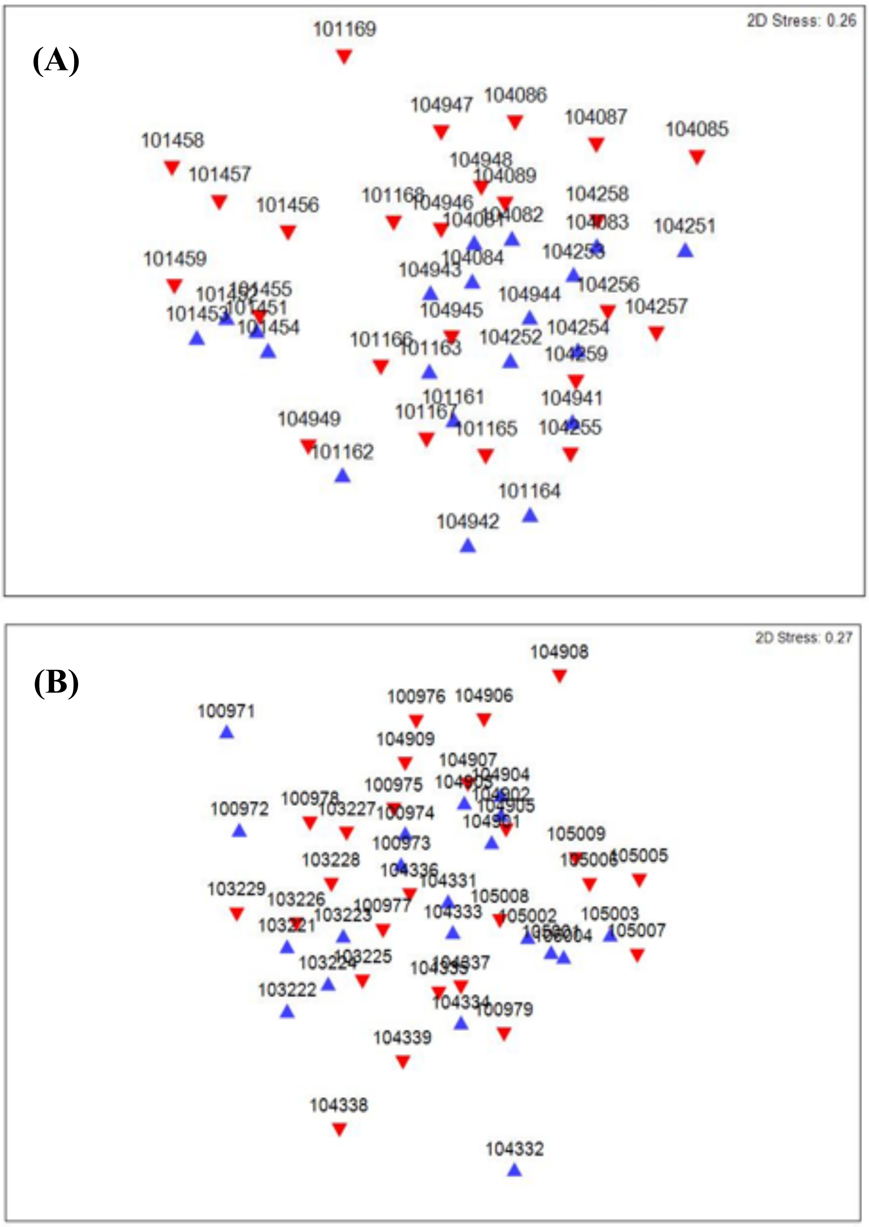

Figure S13 Clustering of gut microbiome of uninfected and infected sheep using ARISA data. (**a**) high-burden; (**b**) low-burden sheep. nMDS plot of sheep based on Bray-Curtis similarity matrix of faecal microbial composition. Blue indicates uninfected whereas red represents infected sheep sample. Sampling were conducted over eight weeks, giving rise to 40 dot points for both high and low burden sheep.

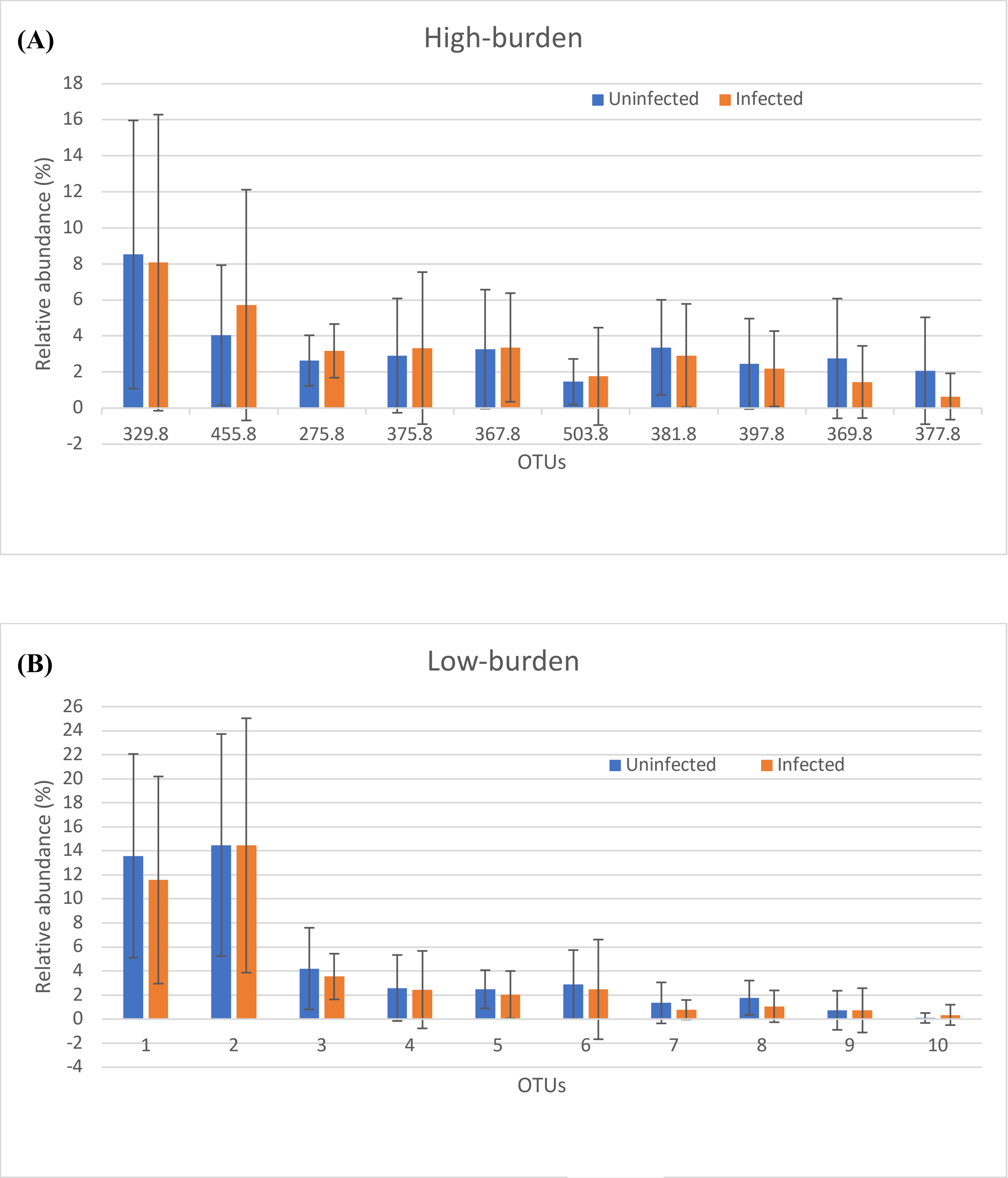

Figure S14 Average relative abundances of commonly detected OTUs of uninfected and infected sheep detected by ARISA. (**a**) high-burden; (**b**) low-burden sheep. The Y-axis shows the average relative abundances (%). X-axis represents OTUs. Error bars represent SD. (Paired t-test; no significance was observed).

Table S1 ANOSIM of microbial composition of sheep between high-burden and low-burden sheep (16S data)

Parameter	Bray-Curtis	Weighted UniFrac	Unweighted UniFrac
R Value	0.878	0.88	0.435
p value	0.001**	0.001**	0.001**

**p ≤ 0.01

Table S2 Identified genera with significantly different abundances between the high and low burden sheep (16S data)

Taxa (genus)	Mean		Median		P value
	High-burden	Low-burden	High-burden	Low-burden	
Unclassified	19.96	21.62	19.94	21.74	0.001***
Unclassified- Bacteroidales	10.54	9.1	10.38	8.93	0.005**
Unclassified- Clostridiales	7.85	10.6	7.63	10.54	0.00004****
Unclassified- Victivallaceae	2.91	1.29	2.97	1.35	0.00002****
Prevotella	2.12	1.62	2.16	1.54	0.04*
Unclassified.RF16	1.7	0.76	1.26	0.7	0.002**
Treponema	1.38	0.8	1.46	0.78	0.004**
Ruminococcus	1.24	1.83	1.2	1.73	0.0005***
Akkermansia	1.23	2.39	1.16	2.17	0.0007***
Unclassified.YS2	1.2	0.56	1.12	0.54	0.0006***
Unclassified- Alpha-Proteobacteria	1.06	0.62	1.12	0.64	0.01**
Unclassified.RFP12	0.8	0.59	0.87	0.65	0.03*
Dorea	0.56	2.13	0.53	2.22	0.0002***
Clostridium	0.37	0.62	0.39	0.66	0.008***
BF311	0.35	0.041	0.29	0.01	0.007***
Unclassified.RF32	0.35	0.27	0.34	0.24	0.03*
Unclassified- Barnesiellaceae	0.23	0.15	0.23	0.12	0.03*
Fluviicola	0.16	0.081	0.14	0.08	0.04*
Unclassified.ML615J28	0.15	0.085	0.14	0.07	0.009**
Unclassified- Erysipelotrichaceae	0.13	0.067	0.12	0.06	0.005**
Geobacter	0.096	0.19	0.085	0.14	0.01**
Unclassified- Christensenellaceae	0.089	0.16	0.095	0.17	0.02*
Unclassified- Enterobacteriaceae	0.068	0.032	0.035	0	0.05*
Unclassified- Flavobacteriaceae	0.051	0.024	0.04	0.015	0.02*
Anaeroplasma	0.033	0.012	0.035	0.005	0.01**
Unclassified- Chroococcales	0.027	0.011	0.02	0.01	0.03*
Unclassified- Dehalobacteriaceae	0.024	0.054	0.03	0.06	0.05*
Pelotomaculum	0.023	0.081	0.02	0.08	0.03*
Unclassified- ML635J21	0.02	0.006	0.015	0.01	0.05*
Unclassified- Anaeroplasmataceae	0.016	0.002	0.02	0	0.01**
Unclassified- Coriobacteriaceae	0.006	0.018	0.005	0.02	0.03*
Salinicoccus	0.005	0	0.005	0	0.01**
Acholeplasma	0.004	0	0	0	0.03*
Cryocola	0.003	0.013	0	0.01	0.03*
Unclassified- Rhodospirillaceae	0	0.027	0	0.025	0.0002***
Coprobacillus	0	0.007	0	0.01	0.005**

Significance determined using the Wilcoxon matched pairs signed rank test

*$p \leq 0.05$, **$p \leq 0.01$, ***$p \leq 0.001$, ****$p \leq 0.0001$

Table S3 The 50 most abundant core OTUs with different abundances between the high and low parasite burden sheep (16S data)

Taxa	Relative abundance (%)	
	High-burden	Low-burden
p__Fibrobacteres__g__Fibrobacter__s__succinogenes_OTU_1	1.495	0.943
p__Bacteroidetes__g__CF231_OTU_2	1.133	1.016
p__Bacteroidetes__g__57N15_OTU_5	0.890	0.803
p__Bacteroidetes__g__Prevotella_OTU_747	0.828	0.629
p__Bacteroidetes__o__Bacteroidales_OTU_11	0.826	0.337
p__Bacteroidetes__o__Bacteroidales_OTU_3	0.824	0.987
p__Bacteroidetes__o__Bacteroidales_OTU_15	0.751	0.527
p__Bacteroidetes__o__Bacteroidales_OTU_9310	0.697	0.773
p__Bacteroidetes__g__57N15_OTU_4	0.686	0.773
p__Proteobacteria__f__Desulfovibrionaceae_OTU_18	0.658	0.579
p__Lentisphaerae__f__Victivallaceae_OTU_31	0.637	0.317
p__Bacteroidetes__f__Rikenellaceae_OTU_23	0.613	0.504
p__Firmicutes__f__Ruminococcaceae_OTU_29	0.538	0.303
p__Bacteroidetes__f__Rikenellaceae_OTU_39	0.528	0.393
p__Bacteroidetes__o__Bacteroidales_OTU_285	0.526	0.33
p__Bacteroidetes__g__Bacteroides_OTU_33	0.499	0.327
p__Firmicutes__o__Clostridiales_OTU_42	0.494	0.602
p__Firmicutes__g__Phascolarctobacterium_OTU_8	0.492	0.524
p__Bacteroidetes__o__Bacteroidales_OTU_82	0.482	0.252
p__Firmicutes__g__Phascolarctobacterium_OTU_7	0.475	0.52
p__Bacteroidetes__o__Bacteroidales_OTU_40	0.472	0.372
p__Spirochaetes__g__Treponema_OTU_16	0.467	0.263
p__Firmicutes__f__Ruminococcaceae_OTU_17	0.454	0.447
p__Bacteroidetes__o__Bacteroidales_OTU_58	0.423	0.301
p__Bacteroidetes__g__57N15_OTU_19	0.420	0.424
p__Firmicutes__g__Oscillospira_OTU_20	0.414	0.399
p__Bacteroidetes__o__Bacteroidales_OTU_35	0.412	0.301
p__Bacteroidetes__f__BS11_OTU_59	0.404	0.296
p__Proteobacteria__c__Alphaproteobacteria_OTU_52	0.399	0.283
p__Bacteroidetes__f__RF16_OTU_28	0.398	0.126
p__Bacteroidetes__o__Bacteroidales_OTU_149	0.386	0.327
p__Firmicutes__f__Ruminococcaceae_OTU_147	0.371	0.325
p__Firmicutes__f__Ruminococcaceae_OTU_80	0.364	0.376
p__Bacteroidetes__f__Bacteroidaceae_OTU_25	0.363	0.331
p__Bacteroidetes__f__Rikenellaceae_OTU_22	0.360	0.366
p__Lentisphaerae__f__Victivallaceae_OTU_168	0.355	0.187
p__Firmicutes__f__Clostridiaceae_OTU_27	0.348	0.352
p__Firmicutes__f__Ruminococcaceae_OTU_72	0.339	0.128
p__Bacteroidetes__g__57N15_OTU_48	0.330	0.400
k__Unassigned_OTU_41	0.326	0.436
p__Bacteroidetes__g__Bacteroides_OTU_47	0.325	0.418
p__Bacteroidetes__o__Bacteroidales_OTU_12	0.324	0.403
p__Lentisphaerae__f__Victivallaceae_OTU_117	0.318	0.157
p__Bacteroidetes__f__RF16_OTU_37	0.308	0.22
p__Firmicutes__o__Clostridiales_OTU_200	0.305	0.209
p__Bacteroidetes__o__Bacteroidales_OTU_152	0.298	0.181
p__Verrucomicrobia__g__Akkermansia_OTU_9	0.297	0.657
p__Bacteroidetes__f__RF16_OTU_65	0.278	0.164
p__Bacteroidetes__g__Prevotella_OTU_30	0.278	0.212
p__Firmicutes__f__Ruminococcaceae_OTU_112	0.277	0.093

Table S4 ANOSIM of microbial composition of high and low parasite burden sheep following infection (16S data)

Parameter	R (Bray-Curtis)	R (Weighted UniFrac)	R (Unweighted UniFrac)
High-burden (n=5)	0.514***	0.629***	0.208**
Low-burden (n=5)	0.233***	0.248***	0.235***

**$p \leq 0.01$, ***$p \leq 0.001$

Table S5 The genera with significantly different abundances between the uninfected and infected sheep with high parasite burden (n=20) (16S data)

Taxa (genus)	Mean		Median		P value
	Uninfected	Infected	Uninfected	Infected	
Unclassified- Bacteroidales	10.78	7.95	10.48	7.93	0.00002****
Unclassified- Clostridiales	7.84	10.12	7.61	10.17	0.0005***
Unclassified- Rikenellaceae	2.56	1.87	2.4	1.85	0.04*
CF231	1.31	1	1.06	0.94	0.04*
Ruminococcus	1.23	2.11	1.19	2.14	0.0003***
Bacteroides	1.19	0.86	1.25	0.82	0.004**
Phascolarctobacterium	0.99	0.86	0.98	0.86	0.04*
Dorea	0.54	1.36	0.48	1.27	0.008**
BF311	0.38	0.09	0.29	0.03	0.01**
Clostridium	0.37	0.51	0.39	0.48	0.02*
Unclassified- ML615J28	0.16	0.09	0.14	0.08	0.01**
Unclassified- Christensenellaceae	0.09	0.15	0.095	0.13	0.05*
Unclassified- Flavobacteriaceae	0.05	0.02	0.04	0.01	0.004**
Unclassified -Desulfarculaceae	0.04	0.015	0.03	0.01	0.007**
Fusibacter	0.02	0.005	0.01	0.005	0.003**
Unclassified- Sinobacteraceae	0.02	0.004	0.01	0	0.006**
Unclassified- Dehalobacteriaceae	0.02	0.05	0.02	0.04	0.02*
Unclassified- SR1	0	0.005	0	0	0.03*
Lactococcus	0	0.006	0	0	0.03*
Candidatus hepatoplasma	0	0.004	0	0	0.03*

Significance determined using the Wilcoxon matched pairs signed rank test

*p ≤ 0.05, **p ≤ 0.01, ***p ≤ 0.001, ****p ≤ 0.0001

Table S6 The genera with significantly different abundances between the uninfected and infected sheep with low parasite burden (n=20) (16S data)

Taxa (genus)	Mean		Median		
	Uninfected	Infected	Uninfected	Infected	P value
Unclassified- Victivallaceae	1.3	0.6	1.36	0.61	0.006**
CF231	1.07	2.75	1.05	1.69	0.008**
Unclassified.S247	0.88	0.56	0.86	0.55	0.02*
Treponema	0.81	1.22	0.79	1.12	0.005**
Unclassified- Chroococcales	0.01	0.002	0.01	0	0.04*
Coprobacillus	0.008	0.001	0.01	0	0.02*
Unclassified- Aeromonadaceae	0.001	0.013	0	0.01	0.05*
Butyricimonas	0	0.007	0	0	0.03*
Succiniclasticum	0	0.1	0	0	0.03*

Significance determined using the Wilcoxon matched pairs signed rank test
*$p \leq 0.05$, **$p \leq 0.01$
